# Deep generative modeling captures maturation-dependent pairing patterns in human antibodies

**DOI:** 10.1016/j.isci.2025.114447

**Published:** 2025-12-22

**Authors:** Lea Brönnimann, Thomas Lemmin, Chiara Rodella

**Affiliations:** 1 Institute of Biochemistry and Molecular Medicine (IBMM), University of Bern, 3012 Bern, Switzerland; 2 Graduate School for Cellular and Biomedical Sciences (GCB), University of Bern, 3012 Bern, Switzerland

**Keywords:** Immunology, Structural biology, Artificial intelligence

## Abstract

Understanding antibody heavy-light chain pairing is critical for decoding immune repertoire architecture and designing therapeutic antibodies, yet most sequence databases lack paired chain information. To address this gap, we developed a two-stage deep learning framework. Transformer-based language models were first pre-trained on large corpora of unpaired heavy- and light-chain sequences, then integrated into a sequence-to-sequence model to generate light chains from heavy chain input. Although native light chain recovery was moderate, generated sequences exhibited high germline identity, improved structural quality, and broader framework and complementarity-determining region coverage. Heavy chains from memory B cells generated light chains with more restricted V gene usage, reflecting maturation-dependent selection. Generated *κ* light chains exhibited a trimodal similarity distribution, indicating distinct functional pairing modes from promiscuous to highly specific. Our approach demonstrates that sequence-to-sequence modeling can uncover inter-chain dependencies and generate plausible antibody pairs, providing a foundation for computational repertoire analysis and therapeutic design.

## Highlights

• A deep generative model enables light chain generation from heavy chains using unpaired data
• Conditioning enhances the structural quality and germline coherence of predicted antibodies
• Memory B cell-derived heavy chains tend to generate light chains with restricted V-gene use
• Generated κ light chains display trimodal similarity, suggesting discrete pairing modes

## Graphical abstract

**Figure:**
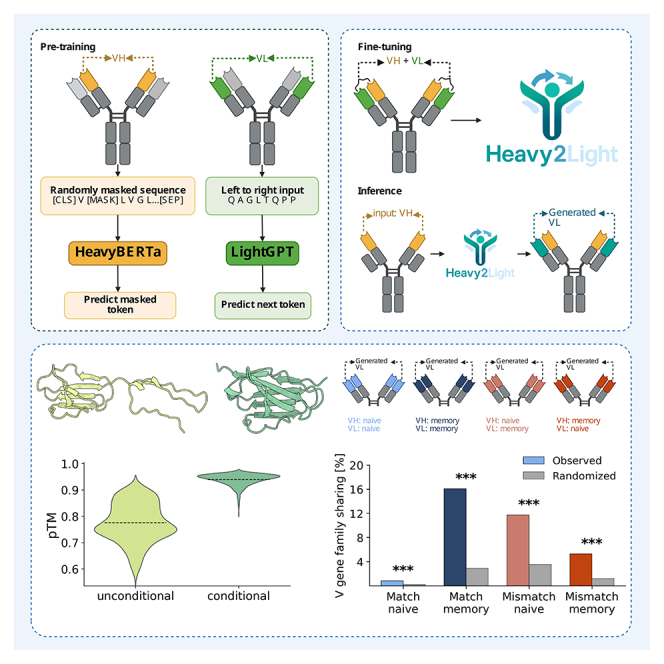

## Introduction

Antibodies are critical effector molecules of the adaptive immune system, produced by B cells to specifically recognize and neutralize pathogenic threats.^1^ The canonical antibody structure consists of two identical heavy chains (HCs) and two identical light chains (LCs), arranged in a characteristic Y-shaped configuration that spatially separates antigen recognition from effector functions.^1^^,^^2^^,^^3^ Antibody LCs belong to one of two classes (*κ* or *λ*), while HCs are classified into five isotypes (IgA, IgD, IgE, IgG, and IgM), with each chain containing constant and variable domains that confer distinct functional roles in the adaptive immune system.^4^ The immense diversity of antibodies results from precise genetic mechanisms, including V(D)J recombination, junctional diversity, and somatic hypermutation.^3^^,^^5^ HC construction involves the selection and joining of V, D, and J gene segments with random non-templated nucleotide additions at junctions, whereas LC rearrangement follows a similar but simpler VJ process that lacks D genes and involves fewer insertions and deletions.^2^^,^^4^^,^^6^ Consequently, LCs are significantly less diverse than HCs, with mounting evidence suggesting that this constraint reflects the evolutionary pressure for LC rearrangements to minimize self-reactivity of B cell receptors.^7^^,^^8^ The variable domains of both HCs and LCs are organized into alternating framework regions (FRs) that maintain structural integrity and complementarity-determining regions (CDRs) that constitute the antigen-binding site, where the six CDRs collectively form the paratope responsible for antigen recognition.^9^^,^^10^ The remarkable combinatorial diversity of antibodies enables recognition of virtually any foreign epitope.^3^^,^^11^ Understanding how this diversity is organized and utilized within antibody repertoires is therefore essential for advancing fundamental immunology, rational vaccine design, and the development of engineered therapeutic antibodies with enhanced specificity and reduced immunogenicity.^12^^,^^13^

The advent of next-generation sequencing (NGS) has revolutionized the study of antibody repertoires, allowing a high-throughput characterization of immune responses at an unprecedented scale.^12^ By sequencing the variable regions of HCs and LCs, researchers can map the diversity and evolution of antibody responses in health and disease. However, NGS generates massive datasets that present significant analytical challenges. Although these technologies can efficiently capture the sequence information from millions of individual B cells, they typically lose the natural pairing information between HC and LC during the sequencing process.^14^^,^^15^^,^^16^ This loss of pairing information creates a substantial knowledge gap, since the function and specificity of an antibody critically depend on the correct association of its HCs and LCs.^17^^,^^18^ Consequently, computational immunology faces a fundamental challenge: how to accurately reconstruct or predict these essential pairing relationships from sequence data alone. The scale and complexity of antibody repertoire datasets further compound this challenge, necessitating advanced computational approaches that can effectively process and interpret vast amounts of immunological data.^12^^,^^19^^,^^20^

Recent advances in deep learning, particularly the emergence of language models, have demonstrated remarkable capabilities in extracting meaningful patterns from large-scale biological datasets.^19^^,^^20^^,^^21^ The application of these models to biological sequences is conceptually grounded in the principle that amino acid sequences encode protein structure and function through positional context and relationships, analogous to how words in sentences convey meaning through order and context.^20^^,^^22^ These models, inspired by natural language processing techniques, have been successfully applied to biological sequences, revealing hidden structures and relationships within repertoires. Although studies on protein and antibody language models have shown substantial progress,^21^^,^^23^^,^^24^^,^^25^^,^^26^^,^^27^^,^^28^ few have explored the specific task of predicting HC-LC pairings and generating one chain given the other.^22^^,^^29^^,^^30^ Such knowledge is important, since certain HCs demonstrate strong preferences for specific LCs, suggesting that the pairing itself can be crucial for the development of stable, efficient therapeutic antibodies.^31^^,^^32^ It has been observed that many antibody pairs naturally favor cognate HC-LC pairing^32^ and that randomly paired HC and LC often result in autoreactive or non-functional B cell receptors.^17^ This preference can also lead to higher yields of correctly assembled bispecific IgG, thereby reducing the need for extensive engineering of antibody fragments to achieve the desired pairing,^32^ which simplifies the development process and enhances the effectiveness and accessibility of therapeutic bispecific antibodies. HC and LC pairing influences antibody structure by affecting the paratope and interface of the variable heavy (VH) and variable LC (VL) domains,^33^ and specific amino acid properties can influence HC-LC compatibility and binding affinity.^34^ Furthermore, recent investigations have demonstrated that memory B cells exhibit a pronounced heavy-light interdependence.^35^ In these cells, the usage of an identical HC V gene is closely associated with the preferential selection of a specific LC V gene, a pattern that is markedly less evident in naive B cells. This suggests that in memory B cells, the HC effectively predicts the optimal LC, highlighting the role of evolutionary pressures in shaping immune responses.

In this study, we leverage deep learning approaches to investigate HC-LC pairing in human antibodies. We develop and evaluate a set of specialized transformer models: HeavyBERTa for HC sequences, LightGPT for LC sequences, classification models for predicting B cell developmental states, and a Heavy2Light encoder-decoder architecture that translates HC sequences into corresponding LC sequences. These models enable us to capture pairing relationships and expand the understanding of repertoire architecture. Our findings provide a framework for applying deep learning to antibody sequence analysis, with implications for both fundamental immunology and therapeutic antibody discovery.

## Results

**Figure 1:**
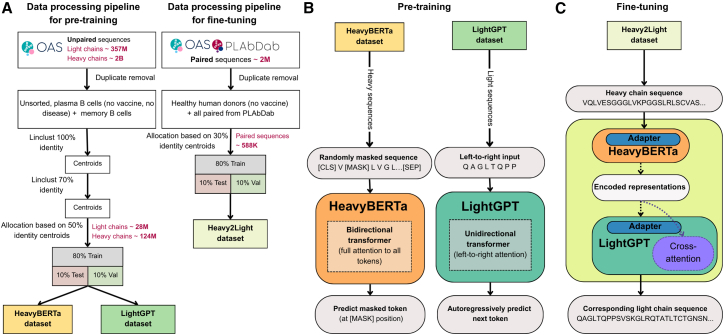
Data processing and training strategy for modeling antibody sequences (A) Data processing pipeline showing the preparation of datasets for the pre-training and fine-tuning of our models. The CDR3 regions (CDRH3 for the HeavyBERTa dataset and CDRL3 for the LightGPT dataset) of unpaired sequences from the OAS (plasma B cells from unsorted samples, memory B cells) are clustered at 100%. Subsequently, the full sequence (heavy or light, for the respective dataset) of resulting centroids are clustered at 70% identity thresholds^23^ and allocated based on 50% centroids to create HeavyBERTa and LightGPT datasets. Paired sequences from OAS and PLAbDab are processed similarly with healthy human donor selection and 30% identity-based allocation, resulting in 80% training, 10% testing, and 10% validation splits for the Heavy2Light dataset. (B) Pre-training phase illustrating the training objectives for each model. HeavyBERTa employs bidirectional transformer architecture with MLM on randomly masked HC sequences, while LightGPT uses unidirectional (left-to-right) transformer architecture for autoregressive next-token prediction on LC sequences. Both models learn sequence representations through their respective self-supervised objectives. (C) Fine-tuning phase demonstrating the encoder-decoder architecture where pre-trained HeavyBERTa (encoder) and LightGPT (decoder) are combined with cross-attention mechanisms and parameter-efficient adapter modules. Only the adapters and cross-attention parameters are trained, while the pre-trained model weights remain frozen. This Heavy2Light model translates input HC sequences into corresponding LC sequences, leveraging the learned representations from both pre-trained components (see also Table S1 for exact dataset sizes; Tables S3–S6 for more information on model architecture).

Understanding HC-LC relationships in antibody repertoires requires models that can capture both individual chain characteristics and inter-chain dependencies. However, paired HC-LC sequences are vastly outnumbered by unpaired sequences, with public databases containing billions of unpaired sequences but only a few million paired examples.^36^ While it is feasible to train a translation model directly on this paired data, the relatively limited size restricts the capacity of large models to generalize effectively and fully capture repertoire diversity.^37^ To address this imbalance and leverage the full extent of available data, we adopted a two-stage modeling strategy. First, we developed two domain-specific language models: HeavyBERTa, based on a masked language modeling architecture for HCs, and LightGPT, a causal language model for LCs. These models were pre-trained separately on more than 99 million HC and 22 million LC sequences from the Observed Antibody Space (OAS) database.^36^ The sequences were restricted to human sequences derived from unsorted B cells, memory B cells, and plasma B cells isolated from peripheral blood mononuclear cells. To eliminate potential bias from immune activation, we exclusively used data from healthy donors with no documented disease or recent vaccination history (Figures 1A and 1B, Table S1).

### Pre-trained models capture V and J gene immunogenetic information

HeavyBERTa was trained with two configurations to predict masked residues within HC sequences. Both variants demonstrated robust performance on this task (Table S2). The larger configuration achieved an accuracy of 89.12%, which was marginally higher than the 88.95% attained by the smaller configuration. In comparison, the LightGPT model yielded comparable results in LC prediction, with an accuracy of 86.35%.

The pretrained models demonstrate a strong ability to capture biologically meaningful features from antibody chain sequences. To examine the structure of these learned representations, we visualized the output embeddings using three complementary techniques: t-distributed stochastic neighbor embedding (t-SNE), principal component analysis (PCA), and linear discriminant analysis (LDA). Across all visualization methods, both HeavyBERTa and LightGPT successfully clustered sequences according to their respective V gene families (Figures S1–S3). Although the t-SNE and PCA projections were predominantly organized by V gene identity, we investigated whether the learned embeddings also captured information about J gene usage. Using LDA, we found clear separability of sequences based on their J gene segments (Figure S3), with t-SNE and PCA visualizations further confirming these clustering patterns (Figures S1 and S2). These results verify that the model preserves immunogenetic information beyond V gene features, with J gene distinctions remaining recoverable through LDA despite the dominant V gene signal.

### Model embeddings distinguish B cell maturation states with high accuracy

Building on these findings, we examined whether the embeddings also reflected B cell developmental states.^38^ B cell maturation is fundamental to understanding immune repertoires, since sequence differences between naive and memory B cells result from somatic hypermutation and selection during affinity maturation.^39^ When LDA was applied to embeddings from both HCs and LCs, we found that both models could effectively distinguish between naive and memory B cell populations (Figure S4). This separation suggests that the models have learned representations that capture biologically relevant differences associated with B cell maturation states. This inherent ability to differentiate B cell states from sequence alone motivated us to fine-tune the HeavyBERTa and LightGPT models for direct classification of sequences according to their B cell origin. The HeavyBERTa model achieved high classification performance, with an overall accuracy of 92.31% and balanced performance across both naive (F1 score, 0.93) and memory (F1 score, 0.92) classes (Table S7). This suggests the model effectively captures sequence features associated with somatic hypermutation patterns. In contrast, the LightGPT model demonstrated slightly lower performance (overall accuracy, 79.17%), with notably stronger identification of memory B cells (recall, 0.90) compared to naive B cells (recall: 0.58).

Our classification results were further validated by examining HC-LC V gene pairing patterns. Memory B cells have been shown to exhibit a pronounced heavy-light interdependence, characterized by enhanced V gene coherence where identical HC V genes preferentially associate with specific LC V genes.^35^ We tested whether this phenomenon would also be observed in previously unlabeled sequences when classified using our HeavyBERTa model. Our results confirmed the expected interdependence phenomenon in memory B cells (Table S8). In the labeled OAS database, memory B cells demonstrated substantially higher V gene coherence (55.82%) compared to naive B cells (13.83%). This pattern was even more pronounced when the HeavyBERTa classifier was applied to previously unlabeled sequences from unsorted B cell populations, where sequences classified as memory origin showed 80.57% coherence versus only 5.60% in those classified as naive origin. When combining all data, memory B cells consistently maintained higher coherence (60.63%) than naive B cells (11.90%). These findings validate both the biological phenomenon of enhanced HC-LC interdependence in memory B cells and demonstrate that our computational model successfully captures sequence features associated with B cell maturation states that correlate with established pairing preferences.

### Heavy2Light captures isotype-specific and maturation-dependent generation patterns

Given the demonstrated ability of our models to capture biologically meaningful patterns and the observed HC-LC interdependencies, we next explored whether these learned representations could be used for sequence generation tasks. To exploit underlying HC-LC pairing relationships, we developed Heavy2Light, an encoder-decoder architecture specifically designed to generate LCs conditioned on HC sequences, thereby explicitly modeling heavy-light interactions. Heavy2Light builds upon the strengths of our individually pre-trained HeavyBERTa and LightGPT models by integrating them into a paired architecture (Figure 1C). To learn the complex HC-LC pairing relationships, Heavy2Light was fine-tuned using a comprehensive dataset of paired antibody sequences. This dataset was compiled from the paired subset of the OAS,^36^ containing sequences from healthy human donors with no recent vaccination history or disease exposure, and the Patent and Literature Antibody Database,^40^ a self-updating repository containing paired antibody sequences curated from patents and academic literature. Starting from an initial pool of approximately 2 million sequences from these combined sources, we clustered the sequences to reduce redundancy (Figure 1), resulting in a final curated dataset of 588′388 paired sequences (Table S1). Fine-tuning was performed using adapter modules,^41^ which enable parameter-efficient training by introducing lightweight, trainable components into the model’s frozen backbone. This approach preserves the valuable pre-trained representations of HeavyBERTa and LightGPT while allowing the model to adapt to the specific pairing task. By updating only a small fraction of the model’s parameters, the use of adapters not only reduces computational requirements but also significantly accelerates training time compared to full model fine-tuning.

**Figure 2:**
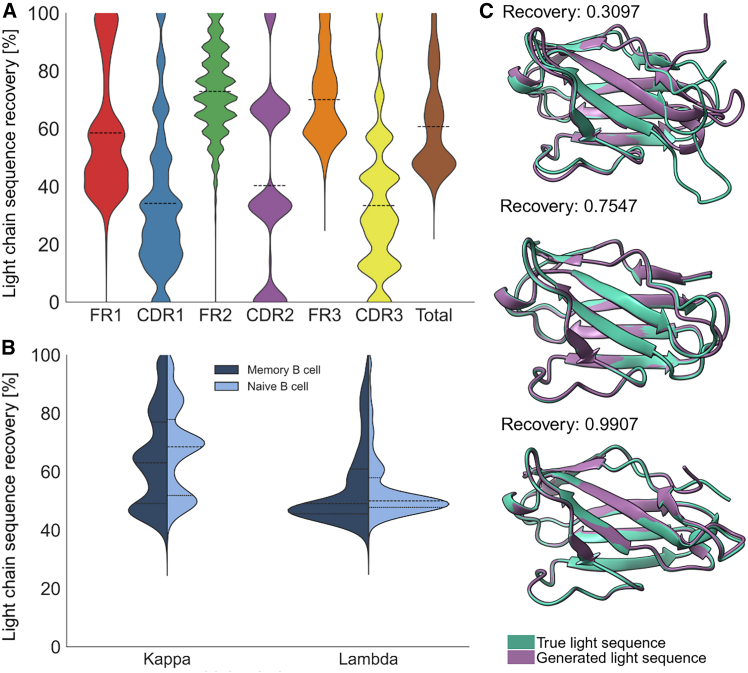
Heavy2Light captures isotype-specific and maturation-dependent generation patterns and maintains structural topology (A) Sequence identity (%) between generated LCs and their corresponding true counterparts across framework regions (FR1–3), CDRs (CDR1–3), and the complete variable domain (*n* = 58,839, Table S9). Black dashed lines indicate the mean values for each region. (B) Sequence identity distributions stratified by B cell maturation state (naive in dark blue and memory in light blue) and LC isotype (*κ* or *λ*, *n* = 53,734). (C) Structural superposition of generated LCs (purple) with their true counterparts (teal) representing three distinct recovery levels. Sequence recovery is given next to each structure.

To assess the quality of our generated LCs, they were aligned with their reference LCs using global alignment. Sequence recovery between generated and true LC pairs shows considerable variation across antibody regions (Figure 2A). FRs demonstrated moderate to high similarity, with FR2 achieving the highest mean identity of 72.90%, followed by FR3 at 70.08% and FR1 at 58.54%. The CDRs showed lower scores, with CDR2 at 40.29%, CDR3 at 33.40%, and CDR1 at 34.15%. Overall, the model achieved a total sequence identity of 60.73% between generated and true LC sequences (Table S9). To uncover additional patterns and gain deeper insights into model behavior, the generated sequences were first classified as memory or naive using our maturation classifier, and subsequently divided them into *κ* and *λ* chains. The sequence identity distributions show distinct patterns across LC types and predicted maturation states. Both *κ* memory and naive B cell-predicted LCs exhibit a trimodal distribution, potentially reflecting underlying structural or functional classes influencing pairing predictability (Figure 2B). Notably, the naive *κ* group exhibits a slight shift toward higher identity values, consistent with its higher mean similarity (67.05%) compared to the memory *κ* group (64.30%). In contrast, *λ* chains display a different behavior: Both *λ* groups lack clear multimodality and are dominated by a peak around 45%. Mean sequence identities were lower across both *λ* groups, with values of 54.91% for sequences predicted to originate from memory B cells and 54.42% for those from naive B cells. To investigate whether these patterns could be attributed to V gene family usage or B cell type origin, we analyzed the distribution across multiple stratifications. Although IGKV1 was overrepresented (Figure S5), neither LC V gene usage, B cell type, nor HC V gene family could explain the trimodal distribution (Figure S6), suggesting that more complex sequence-structure relationships underlie the observed trends. IGKV1 remained the most dominant V gene family across all generated LC sequences, followed by IGKV3, regardless of whether the HCs and LCs were predicted to have matching or non-matching maturation states. Specifically, IGKV1 accounted for 20.2% of sequences in the matching group and 11.9% in the non-matching group, while IGKV3 represented 15.7% and 12.2%, respectively (Figure S7). This pattern was consistent with the distribution observed in the true LCs, where IGKV1 was the most frequent gene family in both the test set (45.03%) and training set (30.58%). IGKV3 was the second most common, comprising 15.07% of the test set and 26.33% of the training set (Figure S7).

### Generated light chains recapitulate germline correlations and maturation-specific pairing rules

Given the moderate sequence recovery, we next evaluated the structural quality of the generated sequences. LCs were folded with Chai-1^42^ and subsequently aligned with their reference sequences with ChimeraX^43^ (Figure 2C). Despite low sequence similarity (as low as 30.97%), the generated LCs aligned well with their true paired counterparts, maintaining correct fold topology. These results suggest that our Heavy2Light model successfully captures the essential structural constraints for antibody folding, prioritizing the preservation of functional fold over the exact reproduction of the sequence. The robust structural conservation across varied sequence similarities demonstrates the model’s ability to generate LCs that maintain the fundamental immunoglobulin framework.

**Figure 3:**
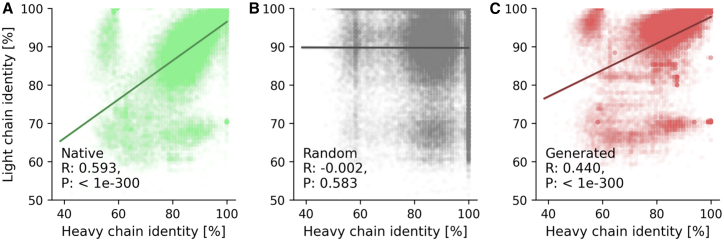
Generated light chains maintain germline identity correlations with heavy chains Germline gene identity to the closest germline V and J gene for both heavy and light chain variable regions (A) Native paired heavy and light chain sequences (*n* = 53,734 pairs). (B) Randomly shuffled heavy and light chains (*n* = 53,734 pairs). (C) Heavy2Light-generated light chains paired with their corresponding input heavy chains (*n* = 53,734 pairs). For each heavy chain, ten light chain sequences were generated by the model; the first sequence matching the maturity status of the input heavy chain was selected for analysis. R: Pearson correlation, P: *p* value.

During B cell maturation, somatic hypermutation introduces mutations into both heavy and LC variable regions.^44^ To assess whether germline identities of paired heavy and LCs show correlated convergence, we calculated the germline gene identity as the percentage sequence identity to the closest germline V and J genes using PyIR, and compared them between heavy and LC pairs for (1) native paired sequences, (2) randomly shuffled pairs, and (3) generated LCs paired with their corresponding input HCs.^30^ Native paired sequences exhibited a Pearson correlation of R=0.593 (P<1e−300) between heavy and LC germline identities (Figure 3A). Randomly shuffled pairs showed no correlation (Pearson R=−0.002, P=0.583, Figure 3B). Heavy2Light-generated LCs exhibited a slightly lower correlation with their paired HCs (Pearson R=0.440, P<1e−300, Figure 3C). Mean germline identity was 89.68% for native LCs, 93.10% for generated LCs, and 86.62% for native HCs.

To further evaluate the biological compatibility of generated LCs with their corresponding HC inputs, we used ImmunoMatch,^29^ a protein language model that estimates the pairing probability between antibody chains. For each input HC, we generated ten LC sequences and used our maturation state classifier to predict whether each sequence was memory or naive. Sequences were then grouped based on whether the predicted maturation state of the LC matched that of the input HC. Sequences with matching maturity states demonstrated superior pairing compatibility compared to non-matching pairs. When HCs and LCs shared the same predicted maturation state, the mean pairing score reached 0.6444, with 76.66% of sequences exceeding the threshold of 0.5. In contrast, sequences with non-matching maturity states achieved a markedly lower mean pairing score of 0.3708, with only 41.57% exceeding the compatibility threshold.

**Figure 4:**
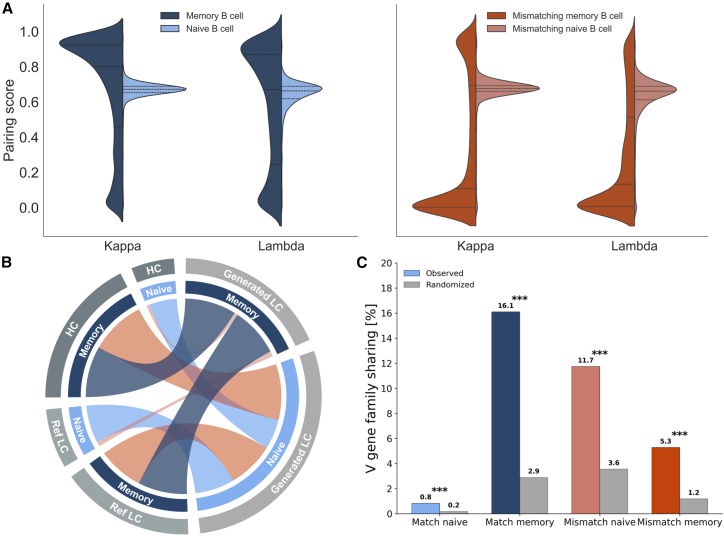
Maturation state concordance drives pairing compatibility and restricts V gene family usage (A) Pairing scores between conditionally generated LC sequences and their corresponding HCs, showing the distribution of predicted pairing affinity across different maturation state combinations (matching maturation: *n* = 53,734, mismatching maturation *n* = 46,081). (B) Chord diagram showing maturation state concordance between input heavy chains (naive: *n* = 138,768; memory: *n* = 449,521), reference light chains (naive, *n* = 186,776; memory, *n* = 401,513), and generated light chains. (C) Proportion of HCs for which ≥80% of generated LCs utilize the same V gene family, comparing observed (colored bars) versus randomized HC-LC pairings (gray bars). Match naive, both HC and LC predicted as naive; match memory, both predicted as memory; mismatch naive, naive HC with memory LC; and mismatch memory, memory HC with naive LC. Ten LCs were generated per HC (*n* = 13,864, 25,694, 392, and 29,321 HCs for match naive, match memory, mismatch naive, and mismatch memory groups, respectively; HCs with <4 sequences excluded). Randomization control with 10,000 permutation iterations per group demonstrates that observed V gene constraints significantly exceed chance expectations. Statistical significance by permutation test: ∗p < 0.05, ∗∗p < 0.01, ∗∗∗p < 0.001 (Table S10).

Among sequences with matched maturation states, naive-naive pairs achieved the highest average score (0.6490), followed closely by memory-memory pairs (0.6425). Despite these similar mean scores, memory-memory pairings displayed a distinct bimodal distribution, with a pronounced secondary peak around 0.9, indicating that a subset of memory-derived pairs achieved exceptionally high compatibility (Figure 4A). In contrast, mismatched pairs demonstrated lower compatibility. Surprisingly, naive HCs paired with memory LCs retained similar pairing ability to the matched maturation groups (average score, 0.6348), whereas memory HCs paired with naive LCs yielded the lowest compatibility (0.3118). Despite pairing scores clearly reflecting the concordance between the predicted maturation states of the heavy and generated LCs, their correlation with sequence recovery, i.e., similarity to the reference LC, was weak for both groups (matching and mismatching maturity pairs, Figure S8).

Although naive HCs were predominantly paired with naive LCs, memory HCs showed a notable bias toward generating LCs predicted as naive. When the input HC was predicted as naive, on average, 90.16% of the generated LC sequences were also predicted to be naive. In contrast, when HCs were predicted as memory B cells, only 48.10% of the generated LCs were predicted as memory (Figure 4B).

We next investigated the interdependence between generated LCs and their input HCs. The consistency of V gene family usage in generated LCs was evaluated. For this, we stratified our data into four groups based on the predicted maturation states of HCs and LCs: (1) both predicted as naive, (2) both predicted as memory, (3) naive HCs paired with memory LCs, and (4) memory HCs paired with naive LCs. Memory B cells exhibited greater V gene family constraint than naive B cells, with 16.1% of memory HCs showing focused V gene family usage (≥ 80% of generated LCs utilizing the same V gene family) compared to only 0.8% of naive HCs (Figure 4C). This pattern extended to mismatched pairings, where memory HCs paired with naive LCs maintained a higher V gene constraint (11.7%) than naive HCs paired with memory LCs (5.3%). Similar V gene constraint patterns were observed across individual V genes as well (Figure S9 and Table S10). To test whether the observed V gene restriction reflects genuine HC-specific pairing constraints or merely recapitulates correlations between maturation state and V gene usage present in the training data, we performed a permutation-based randomization control. By shuffling HC-LC pairings within each maturation state group while preserving the overall V gene distribution, we broke any model-learned associations between specific HCs and their generated LCs. The randomized pairings exhibited significantly lower V gene restriction than the observed pairings across all groups (*p* value = 9.999000e−05, Table S10), demonstrating that the observed constraints exceed background correlations expected by chance.

### Conditional generation enhances structural quality and sequence completeness

**Figure 5:**
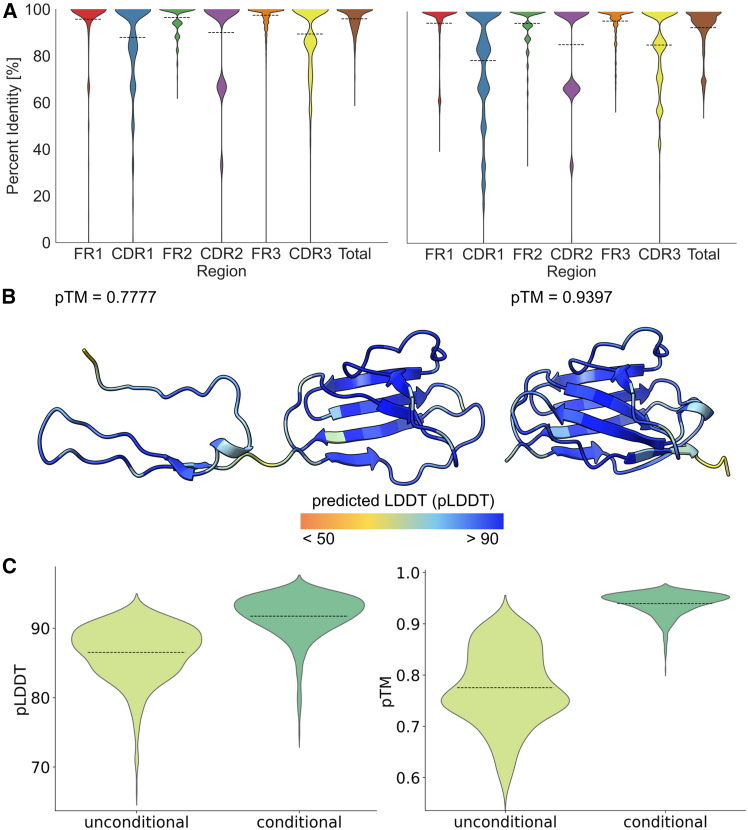
Conditional generation enhances structural completeness and folding quality (A) Germline identity across framework regions (FR1–FR3), complementarity-determining regions (CDR1–CDR3), and complete variable domain (*n* = 53,734, Table S11). (B) Predicted structures for randomly sampled sequences (For A and B, images on the left side show unconditional generation, and on the right side show conditional generation). (C) Predicted structural quality metrics (pLDDT and pTM score) for both generation modes (unconditional, *n* = 705; conditional, *n* = 683).

The conditioning strategy implemented in the Heavy2Light model not only strengthened sequence-structure relationships but also improved the biological plausibility of the generated LCs. When conditioned on a paired HC, the model produced LCs with high germline similarity across all antibody regions (Figure 5A, right). FRs retained high germline identity, with FR3 achieving 95.96%, FR1 at 95.01%, and FR2 at 94.95%. The CDRs achieved slightly lower germline similarity, with CDR2 at 85.76%, CDR3 at 85.62%, and CDR1 at 78.88%. Overall, conditionally generated sequences achieved a total germline similarity of 93.10% (Table S11). In comparison, unconditionally generated sequences (Figure 5A, left) showed slightly higher germline similarities, with FRs consistently exceeding 95% (FR3 at 97.39%, FR2 at 96.39%, and FR1 at 95.68%) and CDRs ranging from 87.90% to 90.00%, resulting in a total germline similarity of 95.86%.

Notably, the Heavy2Light model produced sequences with enhanced structural completeness compared to unconditioned generation. The total sequence length increased to a mean of 96.20 amino acids, representing a substantial improvement over the 80.59 amino acids observed in unconditioned sequences. This extension occurred across all regions: FR1 (25.52 vs. 20.84), FR2 (17.01 vs. 16.64), FR3 (36.00 vs. 32.21), CDR1 (7.39 vs. 6.87), CDR2 (3.03 vs. 3.02), and CDR3 (7.24 vs. 7.10) amino acids (Table S11). The germline similarities, as well as the mean lengths of each region and in total, are similar to the distributions of sequence identity and mean lengths of the training and test set (Figure S10 and Table S12). Importantly, structural folding revealed greatly improved folding quality for Heavy2Light-generated sequences (Figures 5B and 5C). The conditionally generated sequences achieved significantly higher predicted mean template modeling (pTM) scores (0.939) compared to unconditionally generated sequences (0.775), indicating substantially improved structural confidence. Similarly, predicted local distance difference test (pLDDT) scores were markedly higher for conditional generation (91.7) versus unconditional generation (86.5), indicating more reliable local structural predictions throughout the sequence. Additionally, the generated LCs exhibited folding metrics comparable to their corresponding reference LCs, both when co-folded with their cognate HCs and when folded individually. However, when heavy and LCs were randomly shuffled to create mismatched pairs, the resulting co-folded structures achieved interface predicted template modeling (ipTM)^42^ scores only marginally lower to those of correctly paired sequences (Figure S11 and Table S13), suggesting that interface prediction confidence alone cannot distinguish correct from incorrect heavy-light pairings.

## Discussion

Accurately modeling antibody heavy-light chain pairing remains a central challenge in immunology, with broad implications for repertoire analysis, antibody discovery, and therapeutic engineering. While recent advances in deep learning enable the extraction of meaningful patterns from large-scale immune sequence data, the scarcity of paired sequences continues to hinder the training of models that capture inter-chain dependencies. This study introduces a two-stage deep learning framework. Initially, two separate language models, HeavyBERTa for HCs and LightGPT for LCs, were pre-trained on large corpora of unpaired antibody sequences to learn chain-specific representations. These models were subsequently integrated and fine-tuned on a comparatively small set of paired sequences using lightweight adapters, enabling the conditional generation of LCs from HC input. This approach offers a scalable solution for modeling inter-chain dependencies, even when paired sequence data are limited.

To evaluate the biological plausibility of these predictions, our classifier was applied to unlabeled paired datasets and assessed V gene coherence across the predicted maturation states. Sequences classified as memory showed greater V gene coherence between HC and LC than those classified as naive, echoing patterns previously reported in experimentally annotated datasets.^35^ Interestingly, the frequency of coherent pairings was even higher than previously reported, probably reflecting dataset biases, such as restricted donor representation and the undersampling of naive compartments.^14^^,^^36^ Memory-classified HCs generated LCs with restricted V gene usage, even when the generated sequences were classified as naive. In contrast, naive-classified HCs yielded LCs with broader V gene diversity, consistent with a more permissive, less selection-constrained repertoire. Randomization controls confirmed that these constraints significantly exceed background V gene correlations in the training data: memory-classified HCs showed 16.1% restricted V gene usage compared to only 2.9% in randomized pairings (p<0.001), while naive-classified HCs showed similar low levels in both observed and randomized conditions. This demonstrates that the model learned genuine maturation-dependent pairing rules rather than merely reflecting training data biases. These results suggest that maturation imposes directional constraints on pairing preferences, which can be learned and reproduced by a generative model. This is further supported by correlations in germline identity between paired heavy and LCs. The Heavy2Light model exhibited significantly stronger correlations (Pearson correlation, P=0.440) compared to randomized pairings (P=−0.002), and performed comparably to the test set (P=0.593), demonstrating that the model captures meaningful pairing relationships beyond simple V gene frequency distributions in the training data.

Although the Heavy2Light model demonstrated modest recovery of true LC sequences, many generated sequences achieved high ImmunoMatch scores. The weak correlation between recovery rate and ImmunoMatch compatibility indicates that the model may capture features relevant to pairing beyond primary sequence identity. Previous studies have shown that specific residues at defined positions can determine heavy–LC pairing modes,^45^ and that point mutations in CDR-H3 or CDR-L3 can reshape VH-VL interfaces through conformational changes without major sequence shifts.^46^ These findings indicate that generated LCs may retain essential structural or functional determinants for pairing, even when they diverge from the native sequence.

The trimodal distribution of sequence similarity observed in generated *κ* LCs across both naive and memory groups could not be explained by V gene usage bias. Instead, this pattern may reflect a biological organization within the *κ* LC repertoire, presenting distinct challenges for sequence prediction. The lowest similarity mode might represent highly promiscuous, largely public LCs capable of pairing with diverse HCs,^16^ while the higher similarity modes likely correspond to more specialized or antigen-driven variants that have co-evolved with their cognate HCs. This spectrum suggests a continuum from broadly compatible, promiscuous LCs to increasingly specific, co-adapted HC-LC pairings. In contrast, *λ* LCs exhibited a dominant low-recovery mode, consistent with previous reports that *λ* chains have greater structural flexibility, primarily due to a longer and more diverse CDR-L3 region^47^ and increased amino acid diversity in both framework and CDR regions compared to *κ* chains.^48^ This expanded conformational and sequence variability likely complicates accurate sequence prediction. Moreover, the VL domain in *λ* chains is less stabilized, and VL-VH interactions differ from those in *κ* chains,^47^ potentially leading to less stereotyped pairing patterns and further hindering inference of LC sequences solely from HC context.

Conditioning LC generation on HC input markedly improved the structural and biological plausibility of the outputs. Conditioned sequences exhibited more complete antibody regions, with improved coverage of both framework and CDR segments. Structure-based metrics further supported these gains, with conditioned LCs achieving higher pLDDT and pTM scores compared to those generated without the context of a HC. Although generated sequences achieved ipTM scores comparable to true paired sequences (Figure S11), randomly paired sequences yielded slightly lower but similar values (Table S13), indicating that interface metrics from general folding models cannot reliably distinguish correct from incorrect pairings. Nevertheless, the improvements in sequence completeness and overall structural quality suggest that the Heavy2Light model captures inter-chain dependencies relevant to productive antibody folding, highlighting the potential of conditional generative modeling for exploring and designing biologically coherent antibody pairs.

This study supports an emerging paradigm in which antibody sequence modeling advances beyond classification or annotation toward generative biology, where models propose novel yet biologically grounded sequences. Coherence in predicted maturation states between heavy and LCs was associated with more compatible pairings, indicating that maturation-aware generation contributes to biologically plausible outputs. Extending generative modeling beyond structural compatibility toward functional endpoints, such as antigen specificity, epitope targeting, or neutralization, will be essential for translational applications. Such models also offer potential for interrogating repertoire dynamics in infection, vaccination, or autoimmunity, thereby opening new avenues for systems-level immunological insights.

This study demonstrates the feasibility of using deep learning to model the interdependent sequence features that govern antibody pairing and maturation. By integrating discriminative and generative approaches, we show that transformer-based architectures can uncover immunologically meaningful constraints and enable conditional antibody design. These findings lay the foundation for future tools that support repertoire analysis, therapeutic engineering, and the broader goals of data-driven immunology.

### Limitations of the study

The Heavy2Light model demonstrated modest recovery of true LC sequences, with a weak correlation between recovery rate and ImmunoMatch compatibility, highlighting the challenge of predicting exact sequences while maintaining pairing compatibility. The observed sequence recovery rates in the CDR regions (mean similarities 34.15 for CDR1, 40.29 for CDR2, and 33.40 for CDR3) reflect the high intrinsic variability of these regions. CDRs exhibit extensive sequence diversity driven by somatic hypermutation and V(D)J recombination, making exact sequence matching a less informative metric for evaluating generative performance in these regions. While this represents reasonable initial performance for a generative model, there remains substantial room for improvement, particularly through the incorporation of structural constraints or epitope-guided sequence-structure co-design that could better capture the functional requirements of CDR pairing.^49^^,^^50^

The complex and heterogeneous patterns of sequence similarity in generated LCs underscore the challenges of accurately modeling antibody pairing, and sequence-level recovery remains modest. Experimental validation is necessary to confirm the functional relevance of generated sequences. The model exhibited a tendency to produce naive-like LCs, reflecting known limitations of current antibody datasets and language models trained on germline-skewed repertoires.^51^ This bias likely results from the prevalence of germline-proximal sequences in public datasets, driven by the dominance of naive B cells in peripheral blood and the persistence of high germline identity in affinity-matured antibodies. Additionally, the absence of demographic information such as gender, sex, and ethnicity in the used databases prevents the assessment of potential biases or performance variations across different population groups, limiting the ability to evaluate model generalizability across diverse human populations.

## Resource availability

### Lead contact

Requests for further information and resources should be directed to and will be fulfilled by the lead contact, Chiara Rodella (rodella.chiara@gmail.com).

### Materials availability

This study did not generate new unique reagents.

### Data and code availability

• All data used in this study are publicly available from the Observed Antibody Space (OAS) database^36^ at https://opig.stats.ox.ac.uk/webapps/oas/ and the Patent and Literature Antibody Database (PLAbDab)^40^ at https://opig.stats.ox.ac.uk/webapps/plabdab/. All datasets used to train, validate and test our model can be accessed at https://doi.org/10.5281/zenodo.17855785. The weights of all trained models have been deposited at https://huggingface.co/leaBroe and are publicly available as of the date of publication. All other data reported in this article will be shared by the lead contact upon request.
• All original code has been deposited at Github under the repository https://github.com/ibmm-unibe-ch/Heavy2Light and is publicly available as of the date of publication.
• Any additional information required to reanalyze the data reported in this article is available from the lead contact upon request.

## Acknowledgments

The project was supported by the Helmut Horten Young Investigator Program 2022 (project ID: 2022-YIG-089).

## Author contributions

L.B. acquired and analyzed data, designed and trained models, and drafted the manuscript with critical revision and final approval. T.L. contributed to study design, article drafting, critical revision, and final approval. C.R. conceived the original idea and participated in study design, data analysis, article drafting, critical revision, and final approval of the manuscript. C.R. conducted this research while working at the University of Bern and is currently employed at Botnar Institute of Immune Engineering.

## Declaration of interests

The authors declare no competing interests.

## Declaration of generative AI and AI-assisted technologies in the writing process

During the preparation of this manuscript, the authors employed generative AI tools, including ChatGPT (OpenAI) and Gemini (Google), for language refinement. These tools were used solely to improve clarity, coherence, and style. All AI-assisted content was carefully reviewed and substantively edited by the authors, who accept full responsibility for the accuracy and integrity of the final submitted work.

## STAR★Methods

### Key resources table

**Table:** 

REAGENT or RESOURCE	SOURCE	IDENTIFIER
**Deposited data**		

Observed Antibody Space Database (OAS)	Olsen et al.^36^	https://opig.stats.ox.ac.uk/webapps/oas/
Patent and Literature Antibody Database (PLAbDab)	Abanades et al.^40^	https://opig.stats.ox.ac.uk/webapps/plabdab/
All datasets used for training, validating and testing our models	This paper	https://doi.org/10.5281/zenodo.17855785

**Software and algorithms**		

Python 3.9	Python Software Foundation	https://www.python.org/
MMseqs2 13.45111	Steinegger et al.^52^	RRID:SCR_022962 https://github.com/soedinglab/MMseqs2
Code for training all models & all analyses	This paper	https://github.com/ibmm-unibe-ch/Heavy2Light
Huggingface Transformers library 4.40.2	Wolf et al.^53^	RRID:SCR_027381 https://pypi.org/project/transformers/
PyTorch 2.5.1	Paszke et al.^54^	RRID:SCR_018536 https://pytorch.org/
Adapterhub Adapters library 0.2.2	Pfeiffer et al.^41^	https://adapterhub.ml/
Chai-1 0.6.1	Chai Discovery^42^	https://github.com/chaidiscovery/chai-lab
PyIR 1.4.1	Soto et al.^55^	https://github.com/crowelab/PyIR

**Other**		

Trained models (LightGPT, HeavyBERTa, Heavy2Light, HC classifier, LC classifier)	This paper	https://huggingface.co/leaBroe
Predicted protein structures using Chai-1 (Figures 2C and 5B)	This paper	https://doi.org/10.5281/zenodo.17855785

### Experimental model and study participant details

All models were trained using human data from the Observed Antibody Space (OAS^36^). Additionally, paired data from the Patent and Literature Antibody Database (PLAbDab^40^) was used for the Heavy2Light fine-tuning dataset. Four different datasets were created: an HC dataset for HeavyBERTa pre-training, an LC dataset for LightGPT pre-training, a paired sequences dataset for Heavy2Light fine-tuning, and another paired dataset for the B cell classification task. For the HeavyBERTa and LightGPT pre-training datasets, human sequences from unsorted B cells derived from peripheral blood mononuclear cells (PBMCs), memory B cells, and plasma B cells were selected. For the OAS data, we selected only sequences from healthy donors with no history of disease or vaccination. For the Heavy2Light fine-tuning dataset, we combined paired sequences from both OAS (human, healthy donors with no recent vaccination history or disease) and PLAbDab databases. To maximize the number of training sequences while adhering to these selection criteria, we included all qualifying sequences regardless of whether they could be traced to specific individuals. Neither OAS^36^ nor PLAbDab^40^ provides demographic information such as gender or ethnicity; consequently, these attributes are not available for our datasets. To reduce sequence redundancy and ensure proper data splits, we implemented a hierarchical clustering strategy for the pre-training datasets. First, CDR3 sequences (CDRH3 for HCs, CDRL3 for LCs) from the OAS database were clustered at 100% sequence identity using MMseqs2’s linclust module (version 13.45111).^52^ Linclust identifies k-mer matches between sequences, selects a center sequence for each k-mer group, and compares sequences only to relevant centers. This reduces comparisons, enabling near-linear time clustering of large datasets.^52^ The centroids from these clusters were then used to extract full-length HC or LC sequences, which were subsequently clustered at 70% sequence identity. This threshold reduces the risk of data leakage from related sequences between data splits, as sequences below 70% identity typically diverge across multiple CDRs.^23^ The 70% identity clusters formed the basis of our training datasets. To prevent highly similar sequences from appearing in both training and test sets, we performed an additional clustering step at 50% sequence identity, yielding 113,503,257 centroids for the HC dataset and 3,751,662 centroids for the LC dataset, and allocated sequences to training, validation, and test datasets based on clusters rather than random assignment. This approach ensures that sequences within the same 50% identity cluster remain in the same dataset split, thereby preventing data leakage and providing more robust model evaluation. The final HeavyBERTa dataset comprised 123,867,780 sequences (99,094,224 training, 12,386,778 validation, 12,386,778 test), while the LightGPT dataset contained 28,278,253 sequences (22,622,602 training, 2,827,825 validation, 2,827,826 test). After duplicate removal, the paired sequences for the Heavy2Light model were clustered at 30% sequence identity, resulting in 52,262 centroids for allocation into training (80%), testing (10%), and validation (10%) splits, following the same cluster-based allocation strategy to maintain sequence independence across datasets. The final Heavy2Light dataset contained 588,388 paired sequences, from which 78,868 sequences were from PLAbDab and the remaining sequences from OAS (470,711 training, 58,838 validation, 58,839 test). After duplicate removal of the full paired sequences, 62% of sequences from PLAbDab were derived from patents, 24.44% from *Homo sapiens*, 6.4% from *Mus musculus*, 2.73% from synthetic constructs, 2.12% from *Macaca mulatta*, and the remaining 2.31% from other sources.^40^ Although PLAbDab contained non-human sequences, the model training remains strongly biased toward human antibody repertoires, as the vast majority of sequences (528,797 total human sequences from OAS and human entries from PLAbDab, representing 89.87% of the total fine-tuning dataset) are of human origin. To create a balanced and non-redundant dataset of naive and memory B cell receptor sequences for classification, we began by collecting all human paired data sequences available from OAS. Due to the limited availability of Memory and Naive B cell derived human paired sequences, we also included sequences of individuals with vaccination history or disease at the time of sequencing. For each antibody chain (heavy and light) separately, we extracted the aligned amino acid sequences and filtered them to retain unique entries. To balance the dataset, we randomly sampled an equal number of naive and memory B cell sequences, using the smaller of the two class sizes as a reference. To minimize sequence redundancy across data splits, we clustered the sequences using linclust with default parameters, processing HCs and LCs separately. 30% sequence clustering information, resulting in 767,089 centroids for HCs and 111,967 centroids for LCs, was then used to perform non-overlapping train, validation, and test splits: all sequences from the same cluster were assigned to the same subset. This strategy mitigates information leakage across splits. The final HC classifier dataset comprised 842,510 sequences (674,009 training, 84,252 validation, 84,249 test), while the LC classifier dataset contained 228,716 sequences (182,973 training, 22,872 validation, 22,871 test), with larger clusters preferentially assigned to the training set.

### Method details

#### Model architecture and training

Two antibody-specific language models based on the Transformer^53^ architecture were pre-trained: HeavyBERTa for HC sequences and LightGPT for LC sequences. For HeavyBERTa, we implemented two RoBERTa-based configurations to evaluate the impact of model size on performance. The small configuration featured a hidden size of 512, an intermediate size of 2048, 4 attention heads, and 4 transformer layers, totaling 13.15 million parameters. We scaled up to a hidden size of 768, an intermediate size of 3072, 12 attention heads, and 12 transformer layers, resulting in 86.06 million parameters. For LightGPT, we utilized a GPT-2 architecture with a hidden size of 768, 12 attention heads, and 12 transformer layers (85.86 million parameters). Both architectures utilized a vocabulary of 25 tokens, comprising 20 standard amino acids plus 5 special tokens. For downstream applications, we employed parameter-efficient fine-tuning using bottleneck adapters^41^ with multi-head and output adapter configurations enabled. The adapters utilized a reduction factor of 16 and Rectified Linear Unit (ReLU) as activation function (Table S3). The Heavy2Light encoder-decoder model combined the pre-trained HeavyBERTa (encoder) and LightGPT (decoder) architectures with cross-attention mechanisms, where only adapter modules and cross-attention parameters remained trainable while pre-trained weights were frozen (Figure 1). Similarly, for classifying HC and LC sequences into naive or memory B cell states, we fine-tuned the respective pre-trained models (HeavyBERTa for HCs, LightGPT for LCs) using the same adapter configuration, adding classification heads while keeping the base model parameters frozen.

Two RoBERTa-based configurations were pre-trained on unpaired HC sequences using MLM. Both models were trained with identical hyperparameters: a learning rate of 5e-5, weight decay of 0.1, batch size of 16, and the AdamW optimizer with linear learning rate scheduling. The small configuration (4 layers, 512 hidden size) was trained for 22 epochs over 136,254,500 training steps, requiring approximately 244 h of computation. The large configuration (12 layers, 768 hidden size) was trained for 9.18 epochs over 56,832,000 training steps, requiring approximately 673 h (Table S4). The GPT-2-based model was pre-trained on unpaired LC sequences using autoregressive language modeling. Training was conducted for 41 epochs over 12,265,000 steps, with a batch size of 16, a learning rate of 5e-5, a weight decay of 0.1, and AdamW optimization with linear scheduling. The model required approximately 606 h of training time (Table S5). The Heavy2Light translation model was fine-tuned using parameter-efficient adapter-based training, combining the pre-trained HeavyBERTa encoder (small configuration) with the LightGPT decoder in an encoder-decoder architecture. Training was conducted for 50 epochs with a batch size of 64, learning rate of 1e-5, weight decay of 0.1, and gradient clipping with a maximum norm of 1.0. We used AdamW as an optimizer with linear learning rate scheduling. For sequence generation, nucleus sampling was implemented with a top-p value of 0.85, temperature of 0.8, and top-k disabled (set to 0). Generation length was constrained to a maximum of 115 new tokens. For each HC, 10 LCs were generated. For the germline similarities and the true LC recovery, we extracted the first generated light sequence, which matched the maturity of the given input HC. For the other analyses, we used all generated LCs. The model was trained for 367,750 steps over approximately 34.9 h (Table S3). Classification models for naive/memory B cell prediction were fine-tuned using the same adapter-based approach on their respective pre-trained base models. The HeavyBERTa classifier was trained for 200 epochs with a learning rate of 3e-6, batch size of 64, maximum sequence length of 150, and dropout rate of 0.1. The LightGPT classifier was trained for 50 epochs with an identical learning rate and batch size but with a higher dropout rate of 0.3. Both models utilized AdamW optimization with weight decay of 0.01 and maintained frozen base model parameters while training only the adapter modules and classification heads (Table S6). All models were trained using the HuggingFace Transformers library (version 4.40.2).^53^ We used Adapters (version 0.2.2)^41^ for the encoder-decoder models to facilitate modular fine-tuning. PyTorch was used as backend (version 2.5.1)^54^ for both the HuggingFace Transformers Library and the Adapters library. Pre-training and fine-tuning were conducted on NVIDIA A100 or H100 Tensor Core GPUs (each with 80 GB of RAM), depending on availability.

#### Downstream analysis

To assess the biological compatibility of generated LCs with their corresponding HC inputs, we applied the recently published ImmunoMatch model,^29^ which estimates pairing probabilities based on sequence features. For each input HC, we generated 10 LCs and predicted their maturation state (naive or memory) using our classification model. Pairings were initially grouped based on maturation state concordance between the HC and generated LCs: (1) Matching (both predicted as naive or both as memory), and (2) Non-matching (discordant maturation predictions). For further analysis, these were further stratified into four subgroups: (1) Matching memory (both HC and LCs predicted as memory), (2) Matching naive, (3) Non-matching memory (memory HC with naive LCs), and (4) Non-matching naive (naive HC with memory LCs). Prior to ImmunoMatch evaluation, all sequences were standardized using PyIR^55^ to trim the variable regions and remove any non-canonical residues.

To investigate HC-LC interdependence patterns, we analyzed paired sequences from the OAS database. We grouped HCs based on their CDRH3 regions and their V gene. We then removed all groups containing only single entries and excluded groups consisting of sequences from only one patient.^35^ For coherence calculation, we applied our trained HeavyBERTa classifier to predict B cell origin (naive or memory) for HC sequences in paired datasets. For each group containing more than one sequence and originating from different patients, we determined whether the LC V gene was identical across all sequences sharing the same HC V gene. Coherence was calculated as the fraction of groups showing identical LC V gene usage within each predicted B cell category. To assess V gene family usage consistency in generated LCs, we performed a frequency-based analysis across four maturation state pairings between heavy and LCs: (1) both predicted as naive, (2) both predicted as memory, (3) naive HC with memory LCs, and (4) memory HC with naive LCs. For each HC in the dataset, ten LC sequences were generated using our model. Each generated LC was annotated with its V gene and V gene family using PyIR.^55^ We then grouped the generated LCs by their corresponding input HC (i.e., all LCs generated from the same HC sequence) and calculated the frequency distribution of V genes and V gene families within each group. For each HC group, we recorded the proportion of sequences sharing the most frequently used V gene or family. An HC was considered to exhibit V gene constraint if ≥80% of its associated LCs used the same V gene or V gene family. This approach allowed us to quantify the extent of V gene restriction across different maturation-state pairings. The ≥80% threshold was chosen to identify cases where V gene usage was strongly focused. Only HCs with a minimum of four associated generated LCs were included.

### Quantification and statistical analysis

#### Evaluation

The generated light sequences were evaluated using alignment-based percentage similarity. The alignment of the generated and true sequences was performed using global alignment with Biopython (version 1.79), employing a gap opening penalty of −10 and a gap extension penalty of −4. Chai-1 (version 0.6.1)^42^ was used for protein structure predictions of the generated and true LCs using multiple sequence alignments. The generated and corresponding true LC were aligned, superimposed, and visualised using ChimeraX (version 1.9).^43^ All dimensionality reduction visualizations were generated using the following parameters and configurations. Embeddings were extracted from the final hidden layer of each pre-trained model, employing mean pooling across sequence positions. PCA, t-SNE, and LDA were performed using scikit-learn (version 1.5.1). T-distributed Stochastic Neighbor Embedding (t-SNE) was applied with a perplexity of 30. LDA supports a maximum of *n*–1 components for *n* classes, so we included three groups (memory, naive, RV + B cells) for dimensionality reduction (Figure S4), but visualized only the naive and memory groups. PyIR (version 1.4.1),^55^ a wrapper for the IgBLAST^56^ immunoglobulin and T cell analyser, was used to calculate the percent identity to the germline. Three datasets were analyzed to assess the co-evolutionary relationship between heavy and light chain germline identities: native paired sequences with unique light chains from our test set (*n* = 53,734 pairs), randomly shuffled pairs, and Heavy2Light-generated light chains paired with their corresponding heavy chains. To establish a baseline for uncorrelated pairs, heavy chains and light chains from the native dataset were randomly shuffled to create artificial pairs (*n* = 53,734 pairs). Heavy chains were paired with randomly selected light chains from different antibodies in the dataset, maintaining the same overall distribution of germline identities while disrupting the natural pairing relationships. For each heavy chain in the dataset, we generated 10 candidate light chain sequences. The first generated sequence that matched the predicted maturity (naive vs. memory) of the input heavy chain was selected for analysis (*n* = 53,734 pairs). Maturity status was determined based on our classifier described in Methods.

#### Statistical analysis

To test whether the observed V gene restriction exceeded background correlations present in the training data, we randomized the assignment of generated LC sequences to HCs while preserving group membership (i.e., light chains remained within their respective maturation state group). We repeated this randomization 10,000 times per group to establish a null distribution of V gene consistency metrics. P-values were calculated using the permutation test correction: p=(b+1)/(m+1), where *b* represents the number of randomized iterations yielding V gene constraint percentages equal to or exceeding the observed value, and *m* is the total number of permutation iterations.^57^ This correction treats the observed data as one possible permutation under the null hypothesis, ensuring non-zero *p*-values. Statistical significance was assessed at three levels: ∗ *p* < 0.05, ∗∗ *p* < 0.01, ∗∗∗ *p* < 0.001. Pearson correlation coefficients and associated *p*-values were calculated to assess linear relationships between heavy and light chain germline identities. Linear regression and statistical analyses were performed using Python 3.9 with SciPy (version 1.13.1) and NumPy (version 1.23.5).
