# Supplementary material for: Deep generative modeling captures maturation-dependent pairing patterns in human antibodies
Source: iScience. 2025 Dec 22;29(1):114447. doi: 10.1016/j.isci.2025.114447 (PMC12814686; doi:10.1016/j.isci.2025.114447)
Supplement: Document S1. Figures S1–S11 and Tables S1–S13 [file mmc1.pdf]

iScience, Volume *29*

Supplemental information

Deep generative modeling captures maturation-dependent pairing patterns in human antibodies

Lea Brönnimann, Thomas Lemmin, and Chiara Rodella

Document S1. Supplemental Information

Supplemental Figures

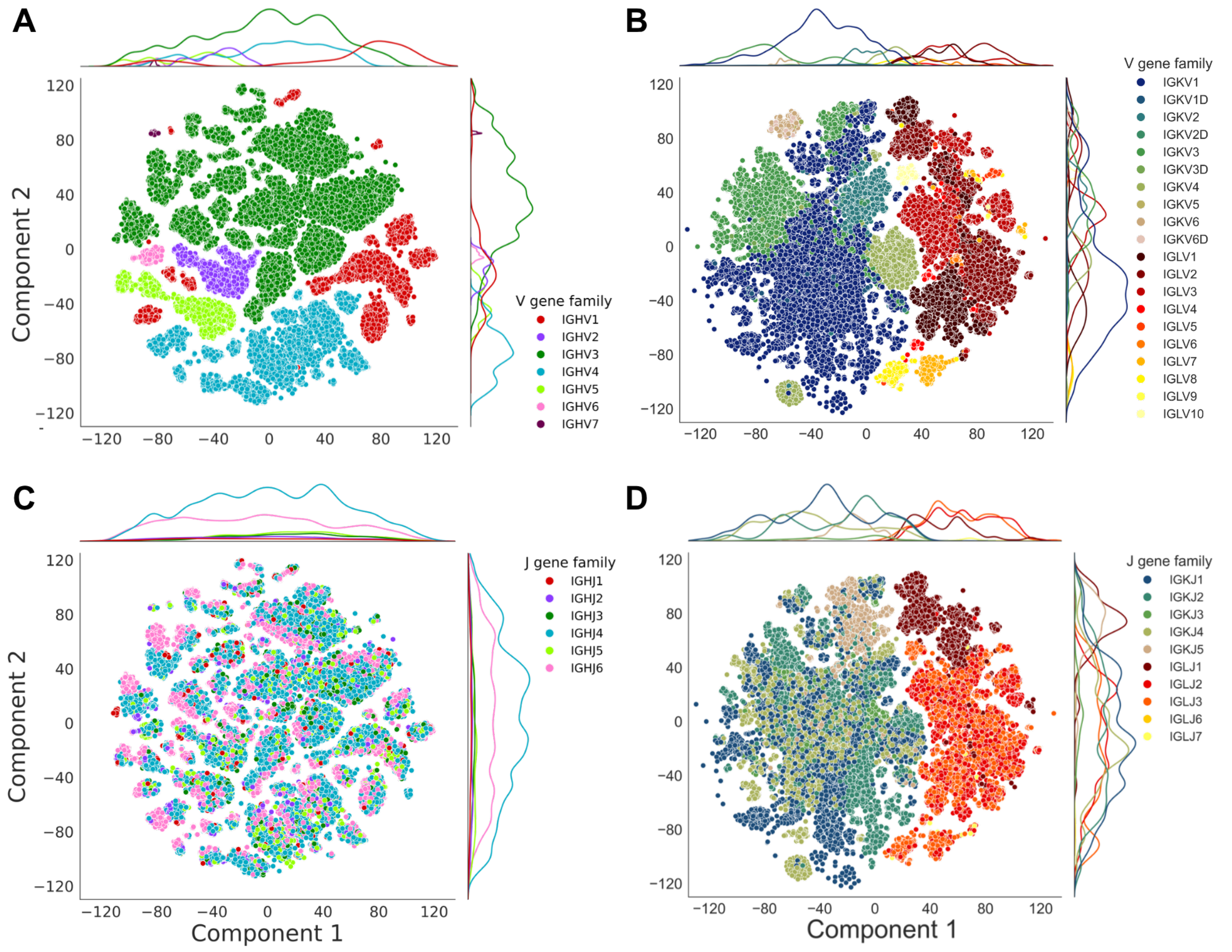

Figure S1: **T-Distributed Stochastic Neighbor Embedding (t-SNE) of final layer embeddings showing V and J gene family clustering in HeavyBERTa and LightGPT models, related to Figure 1**. (A) V gene families from HeavyBERTa model. (B) V gene families from LightGPT model. (C) J gene families from HeavyBERTa model. (D) J gene families from LightGPT model. Each point represents an individual paired sequence (n = 52,138), colored by gene family assignment (κ gene families displayed in cool colors and λ gene families in warm colors). Density plots along each axis show the distribution of sequences across principal components.

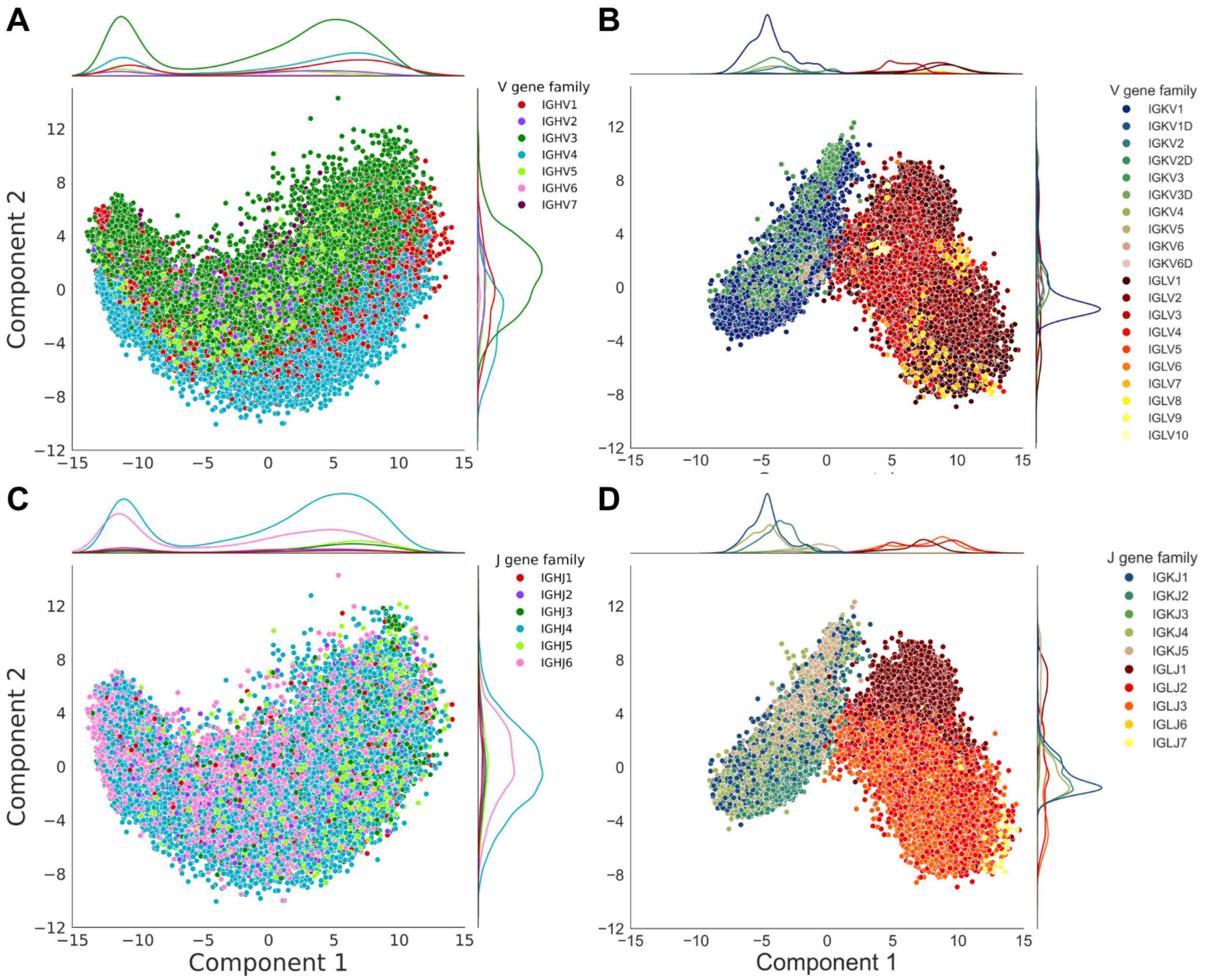

Figure S2: **Principal Component Analysis (PCA) of learned embeddings showing gene family clustering in HeavyBERTa and LightGPT models, related to Figure 1**. (A) HeavyBERTa embeddings colored by HC V gene families. (B) LightGPT embeddings colored by LC V gene families. (C) HeavyBERTa embeddings colored by HC J gene families. (D) LightGPT embeddings colored by LC J gene families. All embeddings are projected onto the first two principal components. Each point represents an individual paired sequence (n = 52,138), with (κ gene families displayed in cool colors and λ gene families in warm colors). Density plots along each axis show the distribution of sequences across principal components.

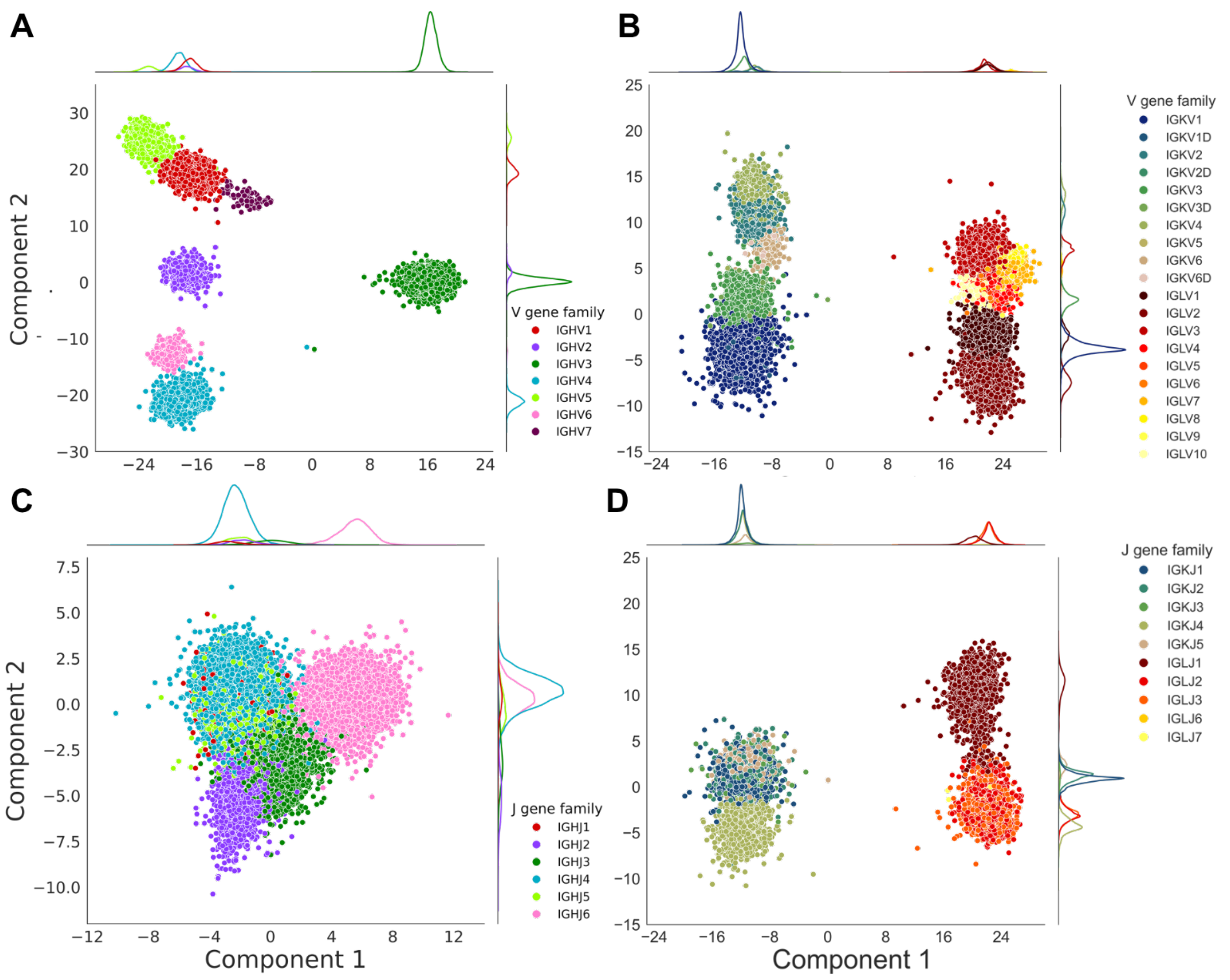

Figure S3: **Linear Discriminant Analysis (LDA) of learned embeddings showing gene family clustering in HeavyBERTa and LightGPT models, related to Figure 1**. (A) HeavyBERTa embeddings colored by HC V gene families. (B) LightGPT embeddings colored by LC V gene families. (C) HeavyBERTa embeddings colored by HC J gene families. (D) LightGPT embeddings colored by LC J gene families. All embeddings are projected onto the first two linear discriminants. Each point represents an individual paired sequence (n = 52,138), with (κ gene families displayed in cool colors and λ gene families in warm colors). Density plots along each axis show the distribution of sequences across linear discriminants.

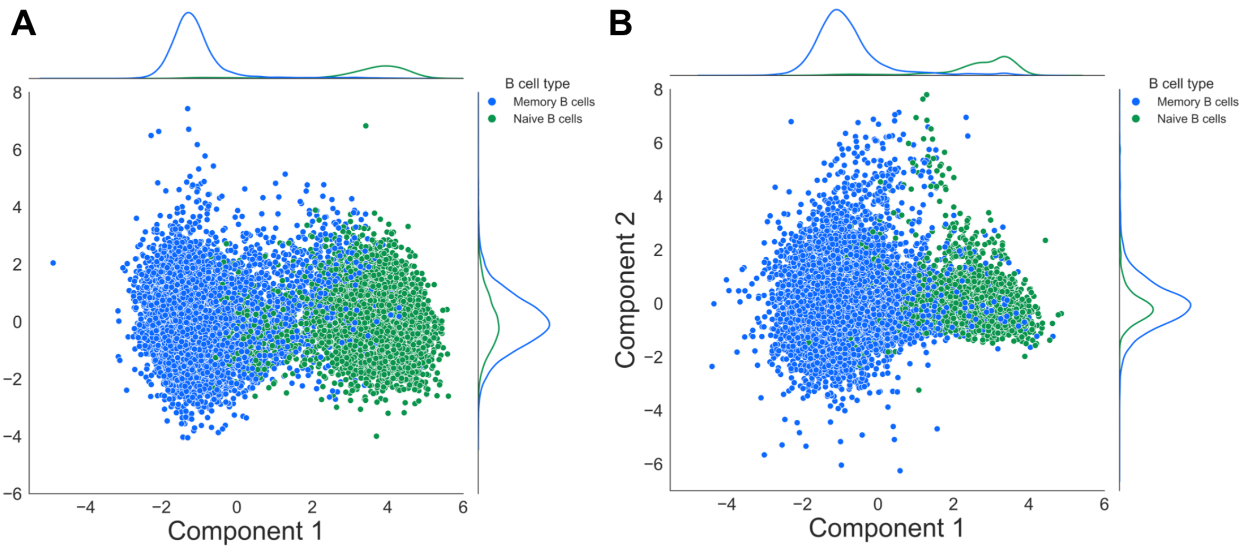

Figure S4: **LDA of final layer embeddings shows separation of memory and naive B cell populations, related to Figures 2 and 4**. (A) HeavyBERTa embeddings projected onto linear discriminants, separating antibody sequences from memory and naive B cell origin (n = 34,558). (B) LightGPT embeddings projected onto linear discriminants, separating memory and naive B cell populations (n = 34,558). The learned representations encode biologically relevant features associated with B cell developmental states.

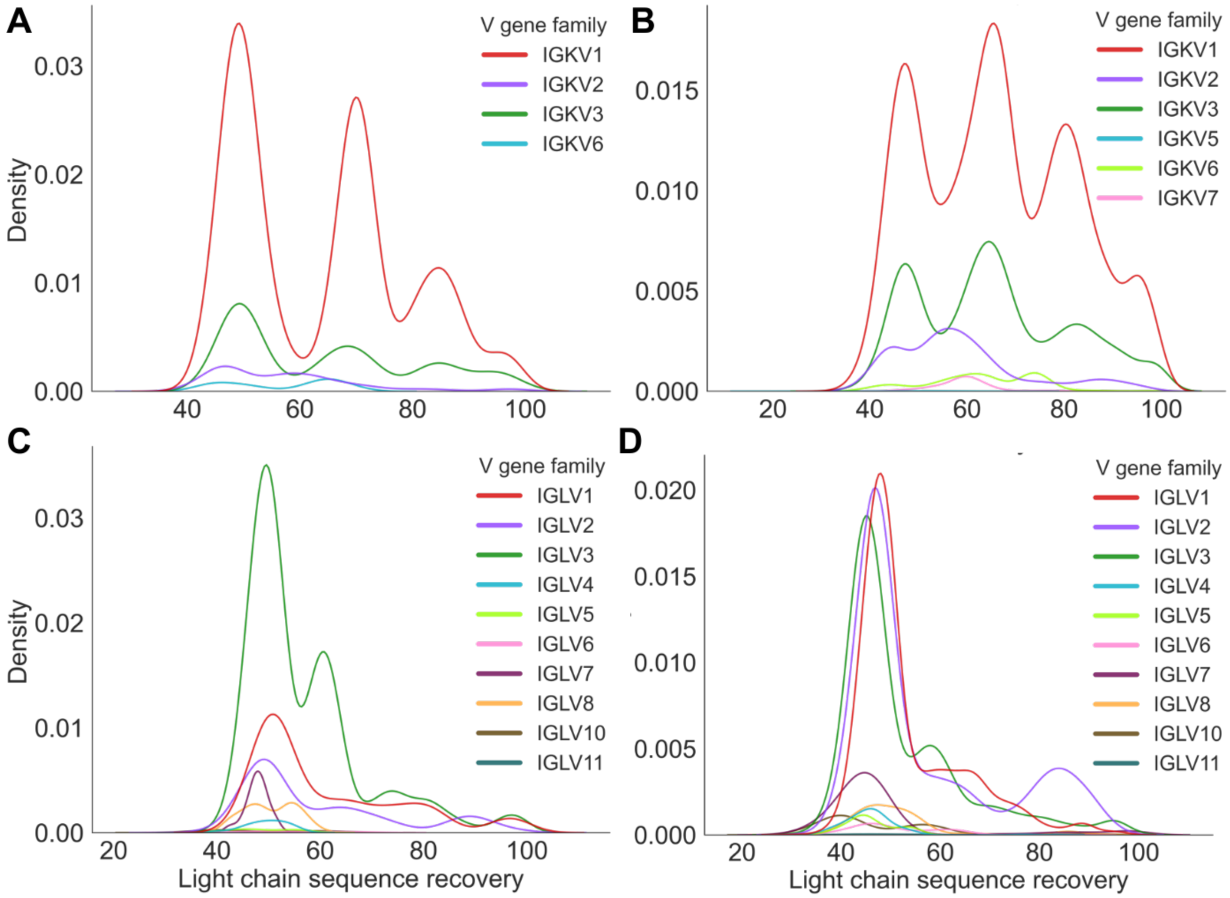

Figure S5: **Light chain (LC) sequence recovery distributions by V gene family for Heavy2Light generated sequences compared to true counterparts across B cell subsets and LC types, related to Figure 2**. (A) κ LCs from naive B cells. (B) κ LCs from memory B cells. (C) λ LCs from naive B cells. (D) λ LCs from memory B cells. Kernel density plots show the distribution of germline identity between Heavy2Light generated LC sequences and their true counterparts, grouped by V gene family (κ sequences: 33,019, λ sequences: 18,261).

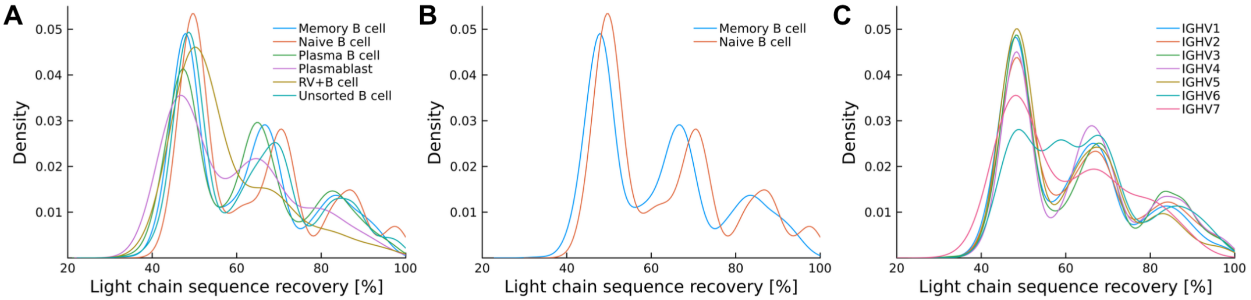

Figure S6: **LC sequence recovery distributions for Heavy2Light-generated sequences compared to native counterparts, related to Figure 2.** (A) Recovery stratified by B cell type. (B) Recovery for memory and naive B cells. (C) Recovery stratified by heavy chain V gene family (n = 52,138).

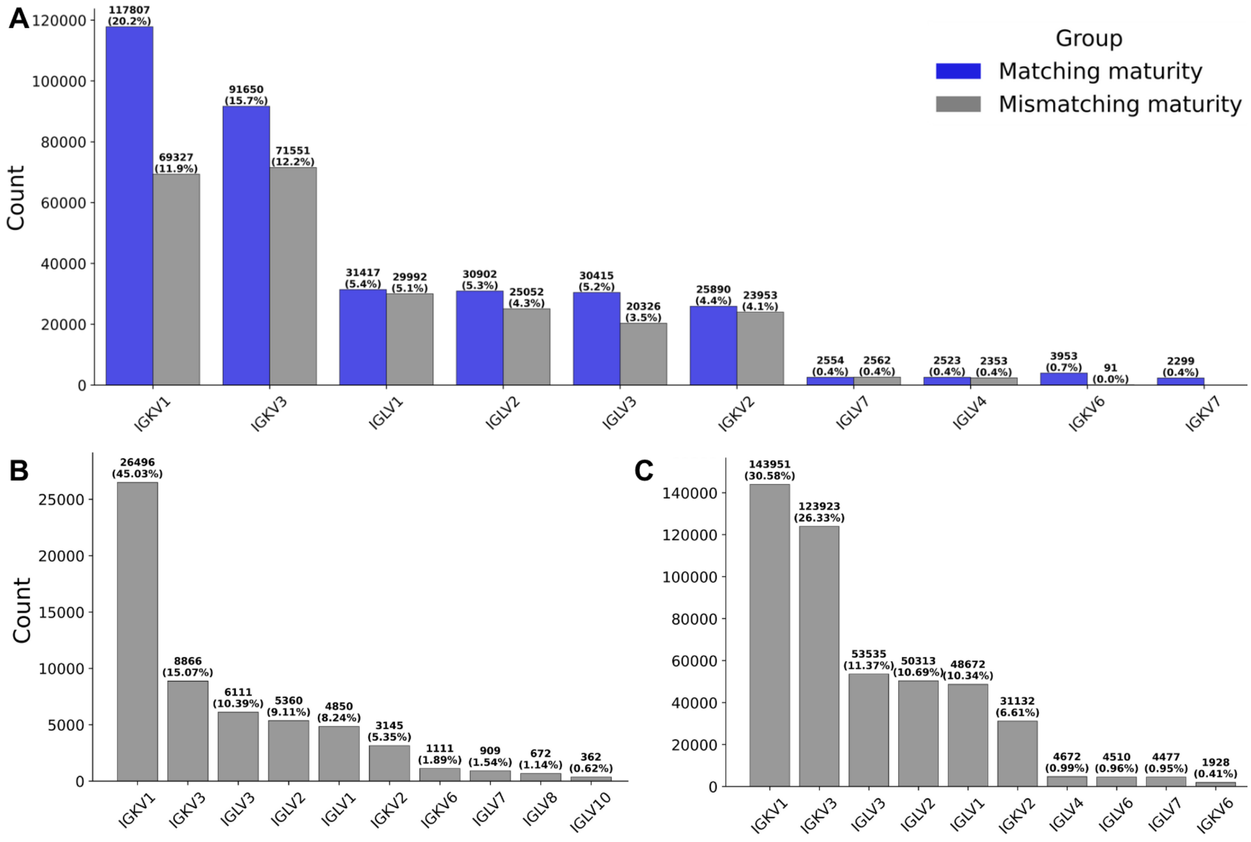

Figure S7: **Comparison of V gene family distributions between Heavy2Light generated sequences and reference antibody LC sequences, related to Figure 2**. (A) V gene family usage in all generated sequences from the Heavy2Light model stratified by matching or mismatching maturity. (B) V gene family usage in true LC sequences from the test set. (C) V gene family usage in true LC sequences from the training set. IGKV1 predominates across all datasets. Numbers above bars indicate absolute counts and percentages.

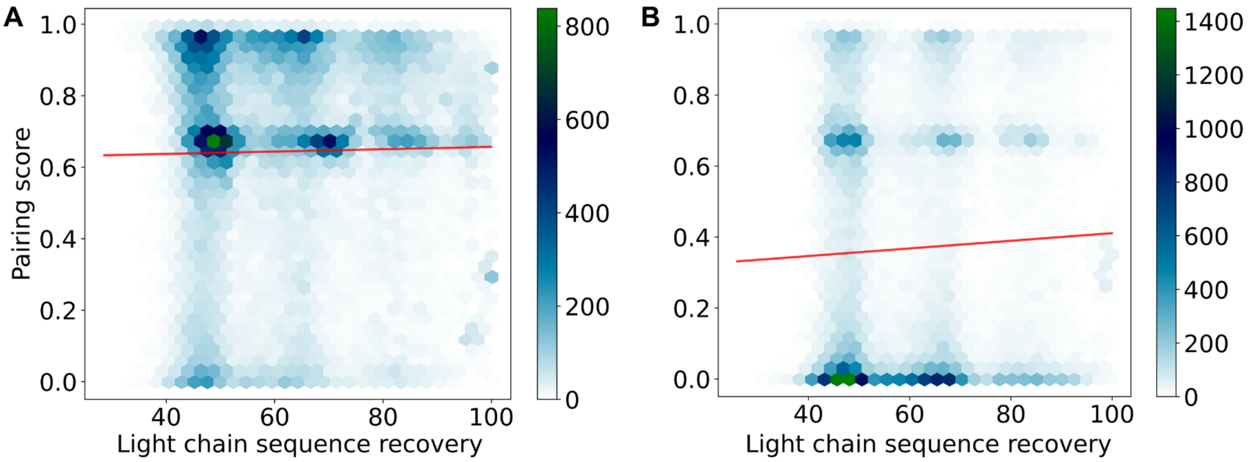

Figure S8: **Correlation between ImmunoMatch pairing probability scores and sequence recovery rates for Heavy2Light generated sequences, related to Figure 4**. (A) Maturity-matched heavy-light chain (HC-LC) pairs, showing HC and generated LCs both predicted to have the same maturation state (memory or naive, n = 53,734). (B) Maturity-mismatched HC-LC pairs, showing different predicted maturation states between heavy and generated LCs (n = 46,082). Scatter plots show the relationship between ImmunoMatch [S1] pairing probability scores and sequence identity between generated and true LC sequences. Maturity-matched pairs show a weak positive correlation (Pearson correlation coefficient $r = 0.0184$, $p = 1.290 \times 10^{-5}$), while maturity-mismatched pairs exhibit a slightly stronger but still weak correlation ($r = 0.0461$, $p = 9.653 \times 10^{-24}$).

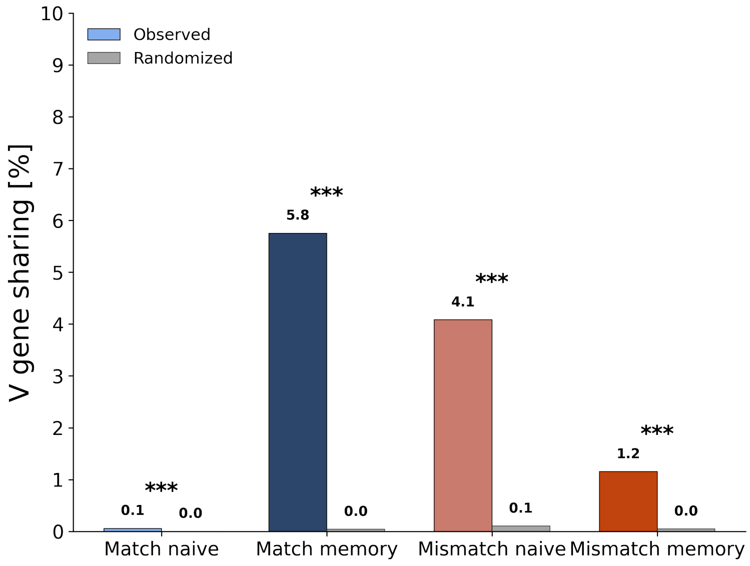

Figure S9: **V gene constraint in Heavy2Light conditionally generated LC sequences across maturity conditions, related to Figure 4**. Proportion of HCs for which ≥80% of generated LCs utilize the same V gene versus randomized HC-LC pairings (gray bars). Match naive: both HC and LC predicted as naive; Match memory: both predicted as memory; Mismatch naive: naive HC with memory LC; Mismatch memory: memory HC with naive LC. Ten LCs were generated per HC (n = 13,864, 25,694, 392, and 29,321 HCs for match naive, match memory, mismatch naive, and mismatch memory groups, respectively; HCs with <4 sequences excluded). Randomization control with 10,000 permutation iterations per group demonstrates that observed V gene constraints significantly exceed chance expectations. Statistical significance by permutation test: *$p < 0.05$, **$p < 0.01$, ***$p < 0.001$.

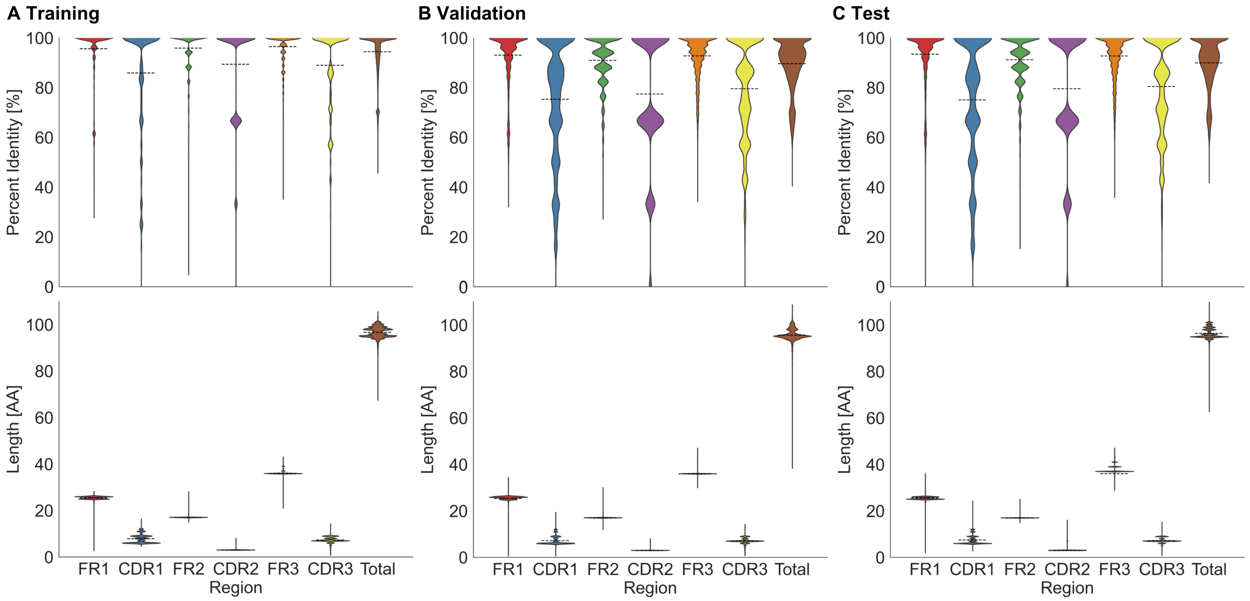

Figure S10: **Distribution of germline identity and region lengths for LC sequences across dataset splits, related to Figure 5**. (A) Training set (n = 470,711). (B) Validation set (n = 58,838). (C) Test set (n = 58,839). For each dataset, violin plots show the distribution of percent identity to germline sequences (upper panel) and sequence lengths (lower panel) for framework regions (FR1-3) and complementarity-determining regions (CDR1-3). Black dashed lines indicate the mean values for each region.

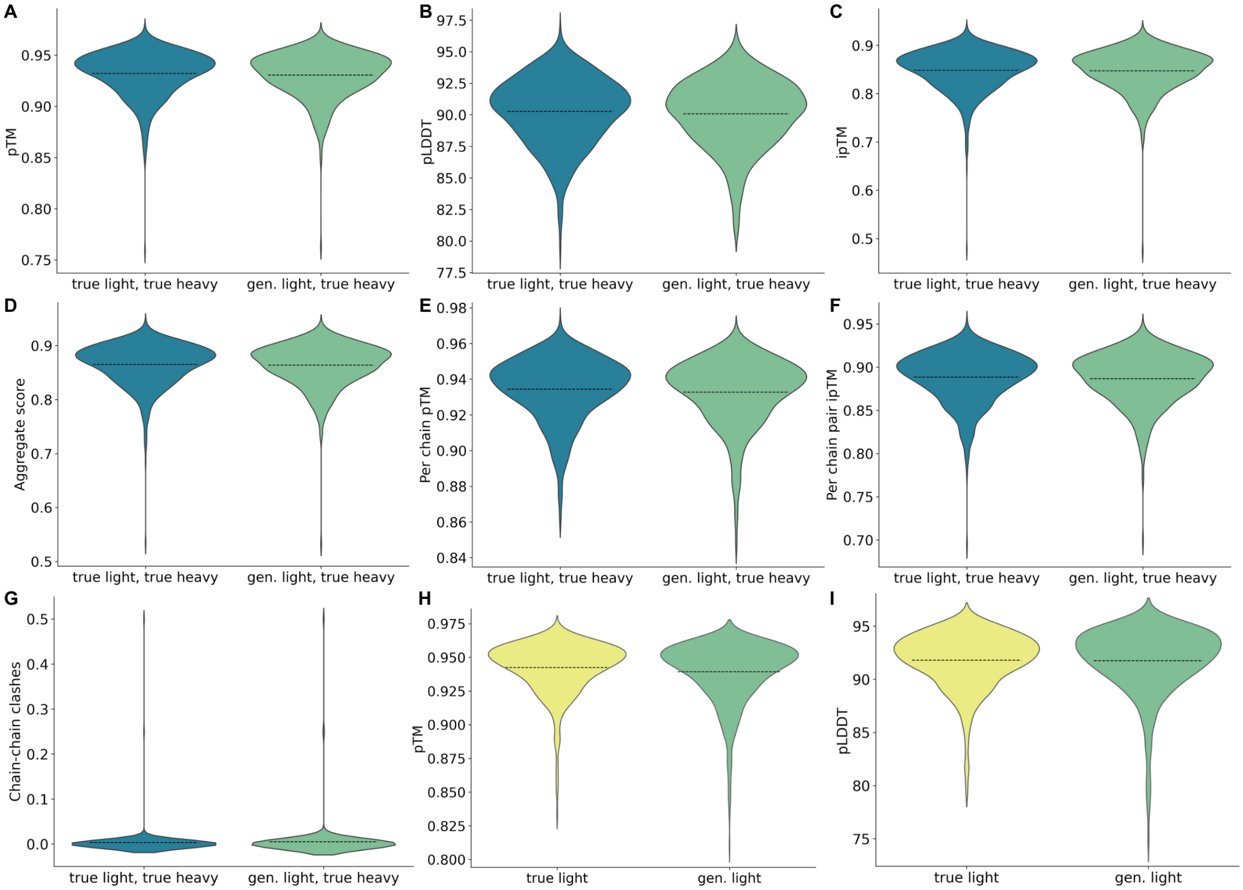

Figure S11: **Structural assessment of heavy-light co-folding and individual LC folding using Chai-1 predictions, related to Figure 5.** (A-G) Comparison of HC-LC co-folding quality between true HC-LC chain pairs (blue) and HCs co-folded with conditionally generated LCs (green) across multiple structural metrics (native HC-LC co-folding: n = 731, co-folding with generated LC: n = 568). (H, I) Individual LC folding quality metrics for individual LC sequences, comparing true LCs (yellow) with conditionally generated LCs (green) folded independently (native LCs: n = 509, generated LCs: n = 682).

Supplemental Tables

	**LightGPT**	**HeavyBERTa**	**Heavy2Light**	**HC classifier**	**LC classifier**
Total clusters (# centroids)	3,751,662	113,503,257	52,262	767,089	111,967
Total sequences	28,278,253	123,867,780	588,388	842,510	228,716
Train dataset (80%)	22,622,602	99,094,224	470,711	674,009	182,973
Validation dataset (10%)	2,827,825	12,386,778	58,838	84,252	22,872
Test dataset (10%)	2,827,826	12,386,778	58,839	84,249	22,871

Table S1: **Dataset composition and data split allocation, related to Figure 1**. Number of sequences and centroids used for training, validation, and testing across all model datasets. HeavyBERTa and LightGPT datasets show unpaired sequence counts with corresponding centroids used for cluster-based allocation (50% identity clustering). The Heavy2Light dataset shows paired sequence counts from combined OAS and PLAbDab sources with centroids derived from 30% identity clustering for allocation across data splits. Datasets for the HC and LC classifiers show paired sequences from the OAS with centroids derived from 30% identity clustering for dataset allocation.

**Model**	**Configuration**	**Antibody chain type**	**Accuracy**
RoBERTa	small	Heavy	0.8895
RoBERTa	large	Heavy	0.8912
GPT-2	–	Light	0.8635

Table S2: **Language modeling performance across model configurations, related to Figure 1**. Accuracy values represent the fraction of correctly predicted amino acid residues for masked language modeling (HeavyBERTa models with 15% random masking) and autoregressive language modeling (LightGPT model with next-token prediction), evaluated on their respective HC and LC test datasets (HeavyBERTa: n = 12,386,778, LightGPT: n = 2,827,826).

Training Configuration	
Number of epochs	50
Batch size	64
Learning rate	$1e^{-5}$
Weight decay	0.1
Optimizer	AdamW
Learning-rate scheduler	Linear
Gradient clipping (max-norm)	1.0
**Model Architecture**	
Model type	Encoder–Decoder
Encoder	HeavyBERTa (RoBERTa-based)
Decoder	LightGPT (GPT-2-based)
Cross-attention	Enabled
Vocabulary size	25
Encoder (HeavyBERTa)	
Hidden size	512
Layers	4
Attention heads	4
Intermediate size	2048
Max sequence length	512
Decoder (LightGPT)	
Hidden size	768
Layers	12
Attention heads	12
Context length	1024
**Generation Parameters**	
Sampling method	Nucleus (top-p)
Top-p	0.85
Temperature	0.8
Top-k	0
Max new tokens	115
**Adapter Configuration**	
Adapter type	Bottleneck
Multi-head adapter	True
Output adapter	True
Reduction factor	16
Activation function	ReLU
**Final Performance**	
Final training loss	0.2078
Final evaluation loss	0.3828
Total training steps	367 750
Training runtime	˜34.9 h

Table S3: **Hyperparameters and performance metrics of the Heavy2Light Encoder-Decoder model, related to Figure 1**. The model combines the HeavyBERTa encoder for HC representation learning with the LightGPT decoder for LC sequence generation.

Hyper-parameter / Metric	Small config.	Large config.
Training setup		
Number of epochs	22	9.18
Batch size	16	16
Learning rate	5×10^{-5}	5×10^{-5}
Weight decay	0.1	0.1
Optimizer	AdamW	AdamW
LR scheduler	Linear	Linear
Model architecture		
Model type	RobertaForMaskedLM	
Vocabulary size	25	25
Max sequence length	512	512
Max position embeddings	514	514
Position embedding type	Absolute	Absolute
Hidden activation	GELU	GELU
Hidden size	512	768
Layers	4	12
Attention heads	4	12
Intermediate size	2048	3072
Performance		
Final training loss	0.4476	0.4379
Final evaluation loss	0.4376	0.4234
Evaluation accuracy	0.8895	0.8912
Total training steps	136 254 500	56 832 000
Training runtime	˜244 h	˜673 h

Table S4: **Hyperparameters and performance metrics for HeavyBERTa model configurations, related to Figure 1.**

Training Configuration	
Number of epochs	41
Batch size / total batch size	16 / 32
Learning rate	5×10^{-5}
Weight decay	0.1
Optimizer	AdamW
LR scheduler	Linear
**Model Architecture**	
Model type	GPT2LMHeadModel
Vocabulary size	25
Context length / max positions	1024
Hidden activation	`gelu_new`
Hidden size	768
Layers	12
Attention heads	12
Inner dimension	3072
Embedding / attention / residual dropout	0.1 / 0.1 / 0.1
Layer-norm ϵ	1×10^{-5}
Initializer range	0.02
**Performance**	
Final training loss	0.3517
Final evaluation loss	0.5446
Evaluation accuracy	0.8635
Total training steps	12 265 000
Training runtime	˜606 h

Table S5: **Hyperparameters and performance metrics for the LightGPT model, related to Figure 1.**

Parameter	HeavyBERTa classifier	LightGPT classifier
Max length	150	150
Batch size	64	64
Epochs	200	50
Learning rate	$3e^{-6}$	$3e^{-6}$
Weight decay	0.01	0.01
Dropout	0.1	0.3

Table S6: **Training parameters for naive/memory B cell classification models, related to Figures 2 and 4.** Hyperparameters used for fine-tuning HeavyBERTa to classify HC sequences and LightGPT to classify LC sequences by B cell developmental state (naive vs. memory).

Metric	HeavyBERTa (HC)			LightGPT (LC)		
	**Naive**	**Memory**	**Overall**	**Naive**	**Memory**	**Overall**
Accuracy	0.9122	0.9367	0.9231	0.5818	0.9044	0.7917
Precision	0.9500	0.8900	0.9242	0.7700	0.8000	0.7887
Recall	0.9100	0.9400	0.9231	0.5800	0.9000	0.7917
F1 score	0.9300	0.9200	0.9232	0.6600	0.8500	0.7838

Table S7: **Classification performance for naive and memory B cell prediction, related to Figures 2 and 4**. Performance metrics for HeavyBERTa and LightGPT models in classifying antibody sequences by B cell developmental state. Metrics are reported for individual classes (naive and memory) as well as overall performance across the entire test dataset (HC classifier: n = 84,249, LC classifier: n = 22,871).

Dataset	Memory B cells	Naive B cells
OAS	0.55817	0.13834
Classified from unsorted B cells	0.80571	0.05595
All combined data	0.60634	0.11190

Table S8: **Coherence percentages of V gene usage, related to Figure 4**. Coherence percentages of V gene usage in memory and naive B cells for the labeled OAS database data (naive: n groups = 253, memory: n groups = 2,338), the previously unlabeled data (naive: n groups = 84, memory: n groups = 350) classified by our model, and all combined data (naive: n groups = 563, memory: n groups = 2,625). The groups are based on identical V gene and CDRH3 sequence, excluding groups of one single individual.

Region	Mean similarity to true light sequence (%)	Length (aa)
FR1	58.5400	25.78
CDR1	34.1521	8.16
FR2	72.8965	17.05
CDR2	40.2847	3.07
FR3	70.0803	36.02
CDR3	33.3994	7.66
Full sequence	60.7300	97.7403

Table S9: **Similarity of generated LC sequences to their corresponding true light sequence, related to Figure 2**. Comparison of identity percentages across antibody regions (FR1–3, CDR1–3, and total sequence) for LCs generated conditionally by Heavy2Light. Values represent mean germline similarity scores to its true light sequence after global alignment; Length indicates the average region length in amino acids (n = 58,839).

Group	Obs. count	Obs. percentage	Rand. percentage	P-value	Sig.
V gene level					
Match naive	8/13,864	0.058	0.001 $\pm$ 0.003	9.999$\times 10^{-5}$	***
Match memory	1,478/25,694	5.752	0.040 $\pm$ 0.012	9.999$\times 10^{-5}$	***
Mismatch naive	16/392	4.082	0.102 $\pm$ 0.161	9.999$\times 10^{-5}$	***
Mismatch memory	338/29,321	1.153	0.046 $\pm$ 0.013	9.999$\times 10^{-5}$	***
V gene family level					
Match naive	114/13,864	0.822	0.175 $\pm$ 0.036	9.999$\times 10^{-5}$	***
Match memory	4,135/25,694	16.093	2.889 $\pm$ 0.099	9.999$\times 10^{-5}$	***
Mismatch naive	46/392	11.735	3.551 $\pm$ 0.868	9.999$\times 10^{-5}$	***
Mismatch memory	1,548/29,321	5.279	1.185 $\pm$ 0.062	9.999$\times 10^{-5}$	***

Table S10: **V gene constraint in conditionally generated light chains with permutation test results, related to Figure 4.** Shows proportion of heavy chains (HCs) with $\geq$80% V gene consistency in generated light chains (LCs), along with statistical validation against randomized HC-LC pairings. Count represents the number of HCs meeting the constraint threshold out of total HCs in each group. Match naive: both HC and LC predicted as naive; Match memory: both predicted as memory; Mismatch naive: naive HC with memory LC; Mismatch memory: memory HC with naive LC. Observed: percentage calculated from counts; Random: mean $\pm$ SD across 10,000 permutation iterations. P-values calculated using permutation test correction: $p = (b+1)/(m+1)$ where b is the number of randomized iterations yielding values $\geq$ observed. ***: $p < 0.001$ [S2].

Region	Germline similarity		Length [AA]	
	**LightGPT**	**Heavy2Light**	**LightGPT**	**Heavy2Light**
FR1	0.9568	0.9501	20.84	25.52
CDR1	0.8790	0.7888	6.87	7.39
FR2	0.9639	0.9495	16.64	17.01
CDR2	0.9000	0.8576	3.02	3.03
FR3	0.9739	0.9596	32.21	36.00
CDR3	0.8934	0.8562	7.10	7.24
Full sequence	0.9586	0.9310	80.59	96.20

Table S11: **Germline similarity and recognized sequence length of generated LC sequences, related to Figure 5**. Comparison of germline identity percentages and amino acid lengths across antibody regions (FR1-3, CDR1-3, and total sequence) for LCs generated unconditionally by LightGPT versus conditionally by the Heavy2Light model. Values represent mean germline similarity scores and sequence lengths in amino acids as determined by IgBLAST alignment (LightGPT: n = 10,000, Heavy2Light: n = 58,839).

Region	Training		Validation		Test	
	**Seq. Id.**	**Length**	**Seq. Id.**	**Length**	**Seq. Id.**	**Length**
FR1	0.9565	25.59	0.9310	25.39	0.9348	25.39
CDR1	0.8595	7.48	0.7536	7.84	0.7510	7.22
FR2	0.9593	17.01	0.9099	17.03	0.9123	17.02
CDR2	0.8944	3.05	0.7749	3.06	0.7958	3.03
FR3	0.9652	36.03	0.9279	36.03	0.9278	36.01
CDR3	0.8902	7.26	0.7960	7.33	0.8049	7.04
Full sequence	0.9448	96.41	0.8967	96.66	0.9000	95.64

Table S12: **Germline identity across antibody regions (FR1–FR3, CDR1–CDR3, and full sequence) for LCs in the training (n = 470,711), validation (n = 58,838), and test sets (n = 58,839), related to Figure 5**. Sequence identity is expressed as the fraction of germline-matched residues; Length is the average region length in amino acids.

Metric	Co-folding			Individual Light Chain	
	**True LC-HC**	**Gen. LC-HC**	**Rand. LC-HC**	**True LC**	**Gen. LC**
Sample size	731	568	933	509	682
Per-chain pTM	0.934	0.933	0.931	—	—
Aggregate score	0.865	0.864	0.848	—	—
pTM	0.932	0.931	0.923	0.942	0.939
ipTM	0.848	0.847	0.829	—	—
Per-chain pair ipTM	0.888	0.887	0.877	—	—
Mean pLDDT	90.27	90.08	89.728	91.79	91.74
Chain-chain clashes	0.003	0.005	0.004	—	—

Table S13: **Structural folding of native and generated LC sequences using Chai-1 predictions, related to Figure 5**. Comparison of folding quality metrics for heavy-light chain co-folding (native HC-LC pairs vs. generated LC paired with native HC vs. randomized native LC paired with HC) and individual LC folding. Values represent mean scores across all evaluated sequences. Co-folding metrics include interface-specific measures (ipTM, per-chain pair ipTM) and chain clashes, while individual LC metrics focus on single-chain folding quality.

Supplemental References

[S1] Guo, Y., et al. (2025). ImmunoMatch learns to predict heavy-light chain pairing from antibody sequence data. *bioRxiv*. doi: 10.1101/2025.02.11.637677

[S2] Phipson, B., & Smyth, G. K. (2010). Permutation P-values should never be zero: calculating exact P-values when permutations are randomly drawn. *Statistical Applications in Genetics and Molecular Biology*, 9(1). doi: 10.2202/1544-6115.1585
